# W Doping in Ni_12_P_5_ as a Platform to Enhance Overall Electrochemical Water Splitting

**DOI:** 10.1021/acsami.1c16755

**Published:** 2021-12-28

**Authors:** Sirshendu Ghosh, Sunil R. Kadam, ShayLee Kolatkar, Alevtina Neyman, Chanderpratap Singh, Andrey N. Enyashin, Ronen Bar-Ziv, Maya Bar-Sadan

**Affiliations:** † Department of Chemistry, Ben-Gurion University, Beer-Sheva 8410501, Israel; ‡ Institute of Solid-State Chemistry UB RAS, 620990 Ekaterinburg, Russian Federation; § Institute of Natural Sciences and Mathematics, Ural Federal University, 620075 Ekaterinburg, Russian Federation; ∥ Chemistry Department, Nuclear Research Centre-Negev, P.O. Box 9001, Beer-Sheva 84190, Israel

**Keywords:** nickel phosphide, γ-NiOOH, DFT calculations, structure-function relationship, oxygen evolution reaction

## Abstract

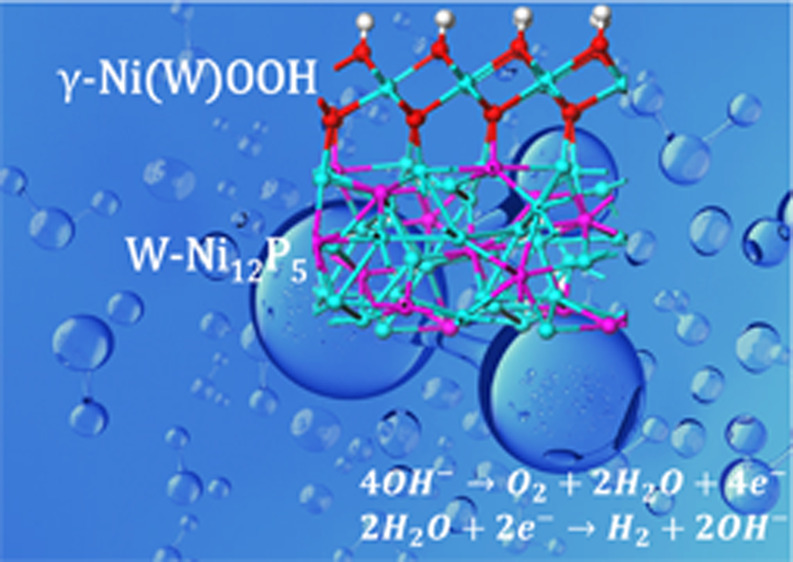

Bifunctional electrocatalysts for efficient hydrogen generation from water splitting must overcome both the sluggish water dissociation step of the alkaline hydrogen evolution half-reaction (HER) and the kinetic barrier of the anodic oxygen evolution half-reaction (OER). Nickel phosphides are a promising catalysts family and are known to develop a thin active layer of oxidized Ni in an alkaline medium. Here, Ni_12_P_5_ was recognized as a suitable platform for the electrochemical production of γ-NiOOH—a particularly active phase—because of its matching crystallographic structure. The incorporation of tungsten by doping produces additional surface roughness, increases the electrochemical surface area (ESCA), and reduces the energy barrier for electron-coupled water dissociation (the Volmer step for the formation of H_ads_). When serving as both the anode and cathode, the 15% W-Ni_12_P_5_ catalyst provides an overall water splitting current density of 10 mA cm^–2^ at a cell voltage of only 1.73 V with good durability, making it a promising bifunctional catalyst for practical water electrolysis.

## 1 Introduction

Hydrogen is considered an ideal renewable energy carrier: it is environmentally benign and has a high gravimetric energy density.^1^ Currently, alkaline water electrolysis is the most attractive way to produce clean hydrogen as a key part of a future energy supply with a zero carbon footprint.^2−4^ The oxygen evolution reaction (OER) is inherently slow compared to H_2_ generation and accounts for most energy losses,^5^ motivating the study of OER catalysts. To date, the need persists for alternative catalysts with lower overpotential and improved stability.

In recent studies, transition-metal phosphides have attracted great interest for their low cost, facile synthesis, and impressive catalytic activity.^6−8^ In an alkaline medium, surface Ni atoms bond with oxygen and hydroxyl groups and are easily transformed into the active NiOOH under a positive bias as a result of the oxidation of Ni^2+^ to Ni^3+^.^9−11^ Previous studies established that NiOOH is the dominant active species in the OER.^9−11^ A further boost in catalytic activity can be achieved by heteroatom doping of NiOOH, for example, with Fe.^12,13^ Another pathway to enhanced activity consists of forming Ni vacancies in Ni(OH)_2_ to reduce the formation energy of the active NiOOH.^14^ Specifically, doping with W is a promising route to boost the activity of the catalyst by enhancing its interaction with intermediates during the reactions.^9,15,16^ Heteroatom doping in transition-metal phosphides (such as V,^17−19^ Mo^20,21^ Mn,^22^ Fe,^23^ and W^24^) is a promising way to improve the electrocatalytic water splitting activity by manipulation of the electronic structure. W doping was shown to improve the electrochemical conductivity and alter the adsorption energy of hydrogen intermediates.^25^ The high affinity of W for water makes it particularly advantageous for alkaline water splitting in particular since the water adsorption and dissociation is a prime step.^25,26^ W doping in NiCoP and Ni(OH)_x_/Co(OH)_x_ showed high electrocatalytic HER activity in alkaline and neutral media attributed to the W capability to accelerate water dissociation as well as to facilitate H_ad_ recombination.^25^

Herein, we used a colloidal thermal decomposition technique to prepare the W-Ni_12_P_5_ phase. The obtained catalysts were used for the electrocatalytic HER and OER in alkaline media. Electrochemical studies show that 15% W-Ni_12_P_5_ displays enhanced catalytic activity performance in 1.0 M KOH, with overpotentials (at 10 mA cm^–2^) of 172 mV with a Tafel slope of 78 mV dec^–1^ for the HER and 322 mV with a Tafel slope 129 mV dec^–1^ for the OER. An amorphous surface layer consisting of WO*_x_*-Ni*_x_*P is formed at higher doping levels and acts as an efficient (pro)electrocatalyst. Using density functional theory (DFT) calculations, we found that for the OER, W doping promotes the formation of high-valence Ni species in a thin shell of γ-NiOOH; such species are known to enhance the OER process. The main role of the W dopants in the γ-NiOOH phase is to reduce the electron density on the adsorbed *O in the rate-determining step of the OER and thus facilitate oxygen evolution. When utilized as both the anode and the cathode, 15% W-Ni_12_P_5_ affords an overall water splitting current density of 10 mA cm^–2^ at a cell voltage of only 1.73 V, with good stability, which opens exciting prospects for practical water electrolysis.

## 2 Experimental Methods

Details of the materials, instrumentation, experimental procedures, and computational methods (DFT) are available in the Supporting Information.

### 2.1 Synthesis of Pure and W-doped Ni_12_P_5_ Nanoparticles

Pure and doped Ni_12_P_5_ nanoparticles were synthesized by the colloidal thermal decomposition technique. For all of the syntheses, oleylamine (OLAM) and trioctylphosphine (TOP) were used as capping agents, with the latter also used as a phosphorus source. For the synthesis of pristine Ni_12_P_5_, 1 mmol of Ni(acac)_2_, 2 mL of OLAM, 3 mL of octadecene (ODE), and 0.3 mL of TOP were mixed in a 50 mL two-neck round-bottom flask. The mixture was heated to 120 °C for 30 min under vacuum in a standard Schlenk line apparatus. The flask was backfilled with dry nitrogen gas and heated to 300 °C at a ramp rate of 15 °C min^–1^, after which the temperature was maintained for 40 min at 300 °C. The product was left to cool to 40 °C by removing the heating mantle. It was then washed with chloroform as the solvent and ethanol as the nonsolvent and was centrifuged for 8 min at 8000 rpm. To ensure complete removal of excess ligands and organic solvent, the washing procedure was repeated at least two times. For the synthesis of W-doped Ni_12_P_5_, tungsten hexachloride (WCl_6_) was added to the reactants at different amounts (0.05, 0.1, 0.15, and 0.2 mM). For doped Ni_12_P_5_, 1 mL of TOP was used. The rest of the protocol was carried out as described above.

### 2.2 Electrocatalytic Measurements for Water Splitting

For the electrochemical experiments, catalyst ink was prepared by mixing 1 mg of the ligand-stripped nanocrystals, 1 mg of carbon black, and 410 μL of Nafion solution (from a mixture of 200 μL of DI H_2_O, 200 μL of isopropanol, and 10 μL of 5% Nafion solution). All of the materials were blended and sonicated for 30 min to form the catalyst ink. The glassy carbon electrodes (3 mm in diameter) were cleaned with alumina micro-polishing powder (0.05 μm) followed by ultrasonication in ethanol and water for 30 s. The homogeneous ink (20 μL) was drop-cast onto a mirror-polished 3 mm glassy carbon electrode to form a final loading of ∼0.7 mg cm^–2^ and was left overnight to dry under ambient conditions. Electrochemical HER and OER measurements were performed in Ar-saturated 1.0 M KOH aqueous solution at room temperature using Ag/AgCl (in KCl) and Hg/HgO (1 M NaOH) as the reference electrode, respectively. All of the electrochemical data were referred to the RHE and were presented as is (i.e., without correction for iR losses).

## 3 Results and Discussion

**Figure 1:**
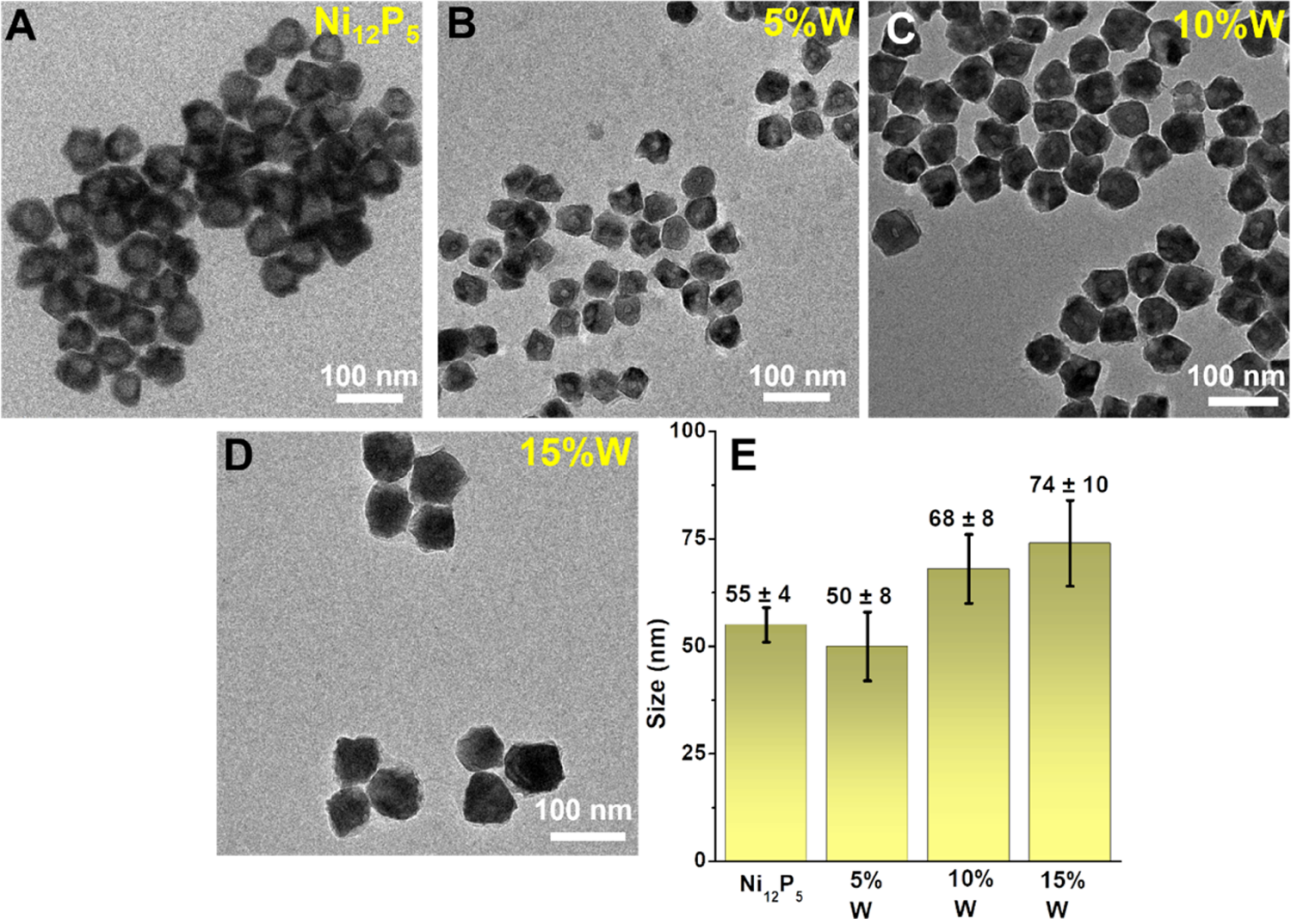
(A–D) TEM images of the as-synthesized nanoparticles and their corresponding size distribution plots (E).

**Figure 2:**
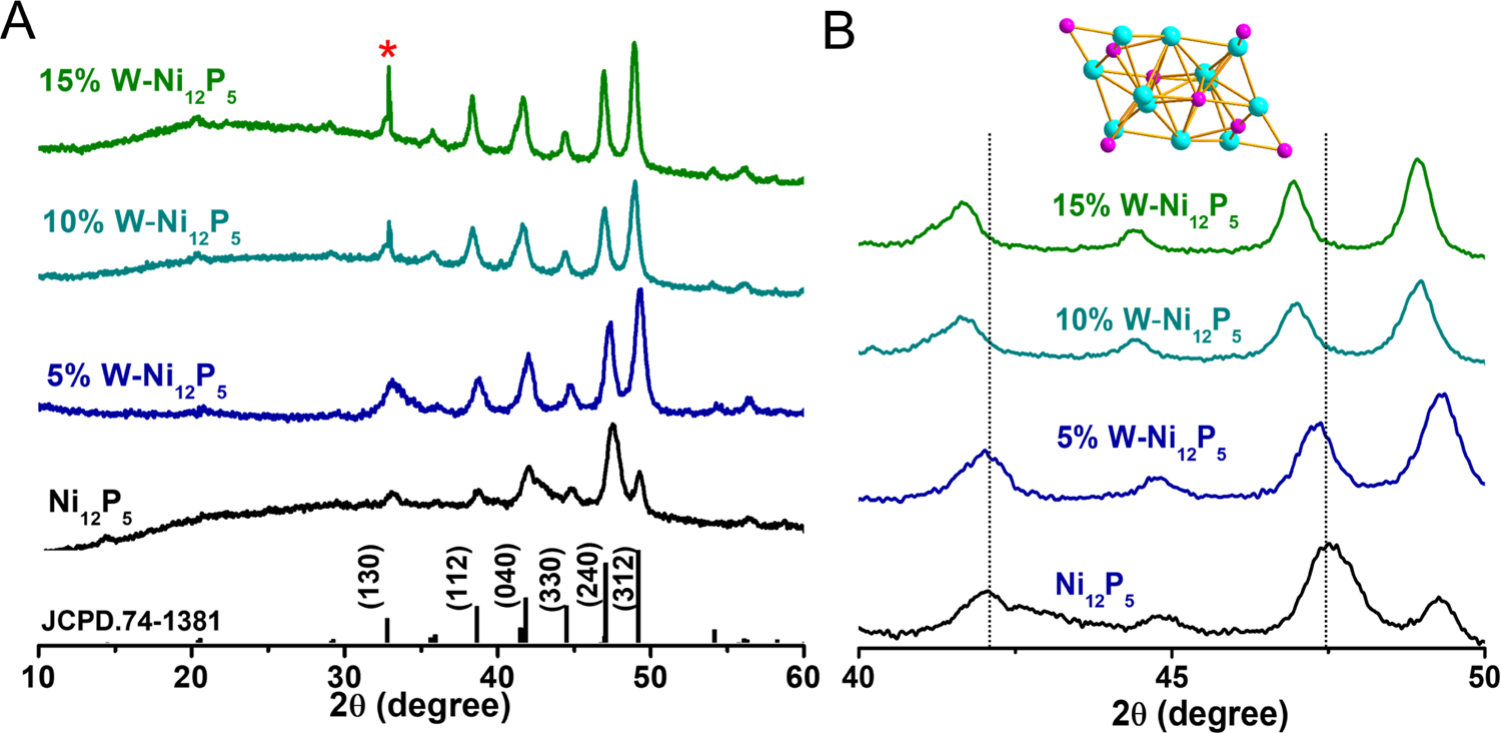
(A) X-ray diffraction (XRD) patterns of the synthesized nanoparticles. The red asterisk refers to the signal from the Si wafer. (B) Enlarged view of the major diffraction peaks in (A), showing a shift toward lower 2θ values. At the top, a ball-and-stick model of the unit cell of tetragonal Ni_12_P_5_ is shown (purple: P, cyan: Ni).

The synthesis of pristine and doped Ni_12_P_5_ structures described in the Experimental Methods section affords truncated hollow polyhedra (Figure 1 and additional images in Figure S2). A general increase in the size and size distribution width was observed upon doping with W (Figure 1E), with increased surface roughness for the samples with high W content (Figures S2 and S3). The homogeneous distribution of the dopants was confirmed by X-ray spectroscopy (EDS) elemental mapping, as shown in Figure S4. The powder X-ray diffraction (XRD) patterns in Figure 2 show that pristine Ni_12_P_5_ is in the tetragonal phase (space group P1(1)-triclinic), which is preserved upon W doping. The crystallographic phase was also confirmed by analyzing high-resolution transmission electron microscopy (HRTEM) images and electron diffraction (Figure S5). The shift in the main diffraction peaks toward lower 2θ values (Figure 2B) was associated with unit cell expansion (see Table S1 and the detailed calculation in the Supporting Information). A unit cell volume expansion of 2.5% occurred by the 15% W doping, in accordance with the DFT calculations (see the Supporting Information).

According to inductively coupled plasma optical emission spectrometry (ICP-OES) measurements (Table S1), the actual content of W in the samples was lower than that expected from the feed molar ratio: 5% in the feed resulted in 0.6% W, and both 10 and 15% feed ratios resulted in ca. 1.7% W in the final material. The XPS analysis below indicated that the surface was enriched with W compared to the bulk, and higher content of surface W correlated with a growing fraction of W in the feed (Table S1).

### 3.1 Electrocatalytic Activity

**Figure 3:**
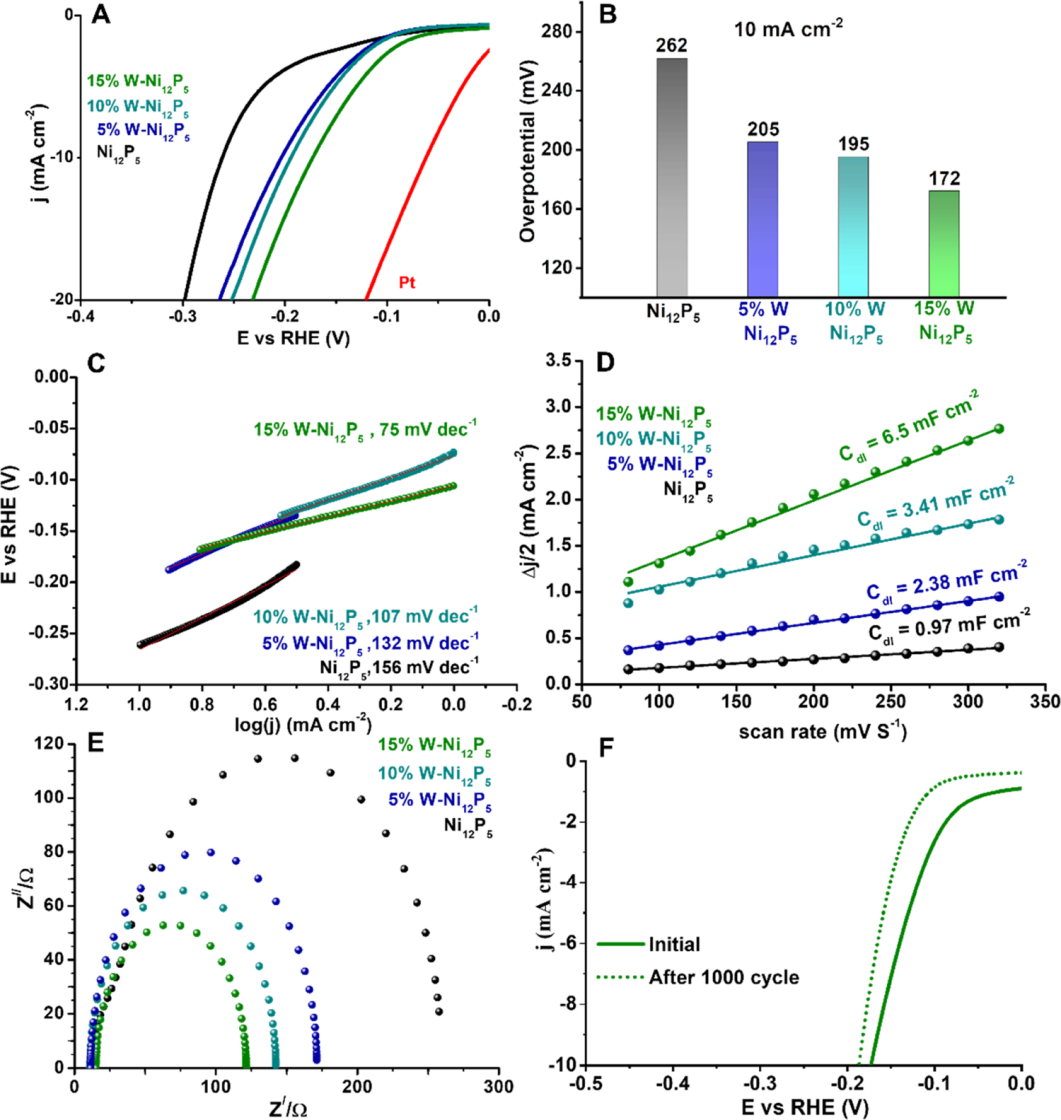
Electrochemical HER performances of pristine and W-doped nickel phosphide catalysts in alkaline solution (1.0 M KOH): (A) LSV polarization curves, (B) overpotentials at a current density of 10 mA cm^–2^, (C) Tafel plots, (D) electrochemically active surface area (ECSA), (E) Nyquist plots obtained at an overpotential of η = 200 mV for the HER. Figure S9 shows the equivalent circuit, and (F) stability test for the 15% W-Ni_12_P_5_ catalyst.

The HER performance of the catalysts in 1.0 M KOH was evaluated by linear sweep voltammetry (LSV) and is presented in Figure 3A without iR-correction and referenced to a reversible hydrogen electrode. The best catalytic activity was observed for 15% W-doped Ni_12_P_5_, with an overpotential of 172 mV to reach 10 mA cm^–2^, compared with 226 mV for the pristine material. The LSV curves were fitted to the Tafel equation (η = *b* log(j) + a, where η is the overpotential, *b* is the Tafel slope, and j is the current density) and are presented in Figure 3C. A significant reduction in the Tafel slope was observed upon doping, from 156 mV dec^–1^ for the pristine material to 75 mV dec^–1^ for the 15% W-doped samples; this highlights the effective acceleration of HER kinetics by the W doping in Ni_12_P_5_. The electrochemically active surface area (ECSA) of the samples, which is proportional to the electrochemical double-layer capacitance (*C*_dl_) of the electrocatalyst, was measured by cyclic voltammetry at various scan rates (Figures S6 and S7).^27,28^ The 15% W-Ni_12_P_5_ showed the largest *C*_dl_ (ca. 6 times higher than undoped Ni_12_P_5_, see Figure 3D), thus increasing the electrode surface accessible to the electrolyte. To distinguish between the contribution of the increased electrochemical active surface from improved intrinsic activity, we plotted the LSV normalized to the ECSA (Figure S8). The normalized curves show an opposite trend of HER activity compared with Figure 3A, which concludes that W doping mostly provides more solution-accessible catalytic sites. This has also been confirmed by DFT calculations of the thermodynamic stability of W-Ni_12_P_5_ solid solutions and the energies for the overall alkaline HER process using Ni_12_P_5_ and W-Ni_12_P_5_ (see more information in the DFT calculations section in the Supporting Information).

To understand the electrode kinetics during the HER process, we carried out electrochemical impedance spectroscopy (EIS) measurements at −200 mV versus RHE. Figure 3E shows the Nyquist plot, where the interfacial charge transfer resistance (*R*_ct_) correlates with the diameter of the semicircular region (equivalent circuit presented in Figure S9). *R*_ct_ gradually decreases upon doping: 15% W-doped Ni_12_P_5_ exhibits an *R*_ct_ of 121 Ω, which is much smaller than that of pristine Ni_12_P_5_ (*R*_ct_ = 258 Ω), indicative of faster interfacial charge transfer kinetics for the W-doped Ni_12_P_5_ catalysts. The lower charge transfer resistance between the catalyst and the electrolyte boosts the sluggish electron-coupled water dissociation process—the Volmer step (H_2_O + e^–^ → H_ads_ + OH^–^)—as revealed by the low value of the Tafel slope of 15% W-doped Ni_12_P_5_ and by the correlation with the DFT data for water adsorption and dissociation on the W-doped Ni_12_P_5_ surface (Figure S23A,B). In addition, the stability of the 15% W-doped Ni_12_P_5_ catalyst was tested by subjecting it to continuous cyclic voltammetry (CV) sweeps at 100 mV s^–1^. Figure 3F shows the LSV plots before and after performing 1000 CV scans at 100 mV s^–1^; only a negligible loss in HER activity was observed.

**Figure 4:**
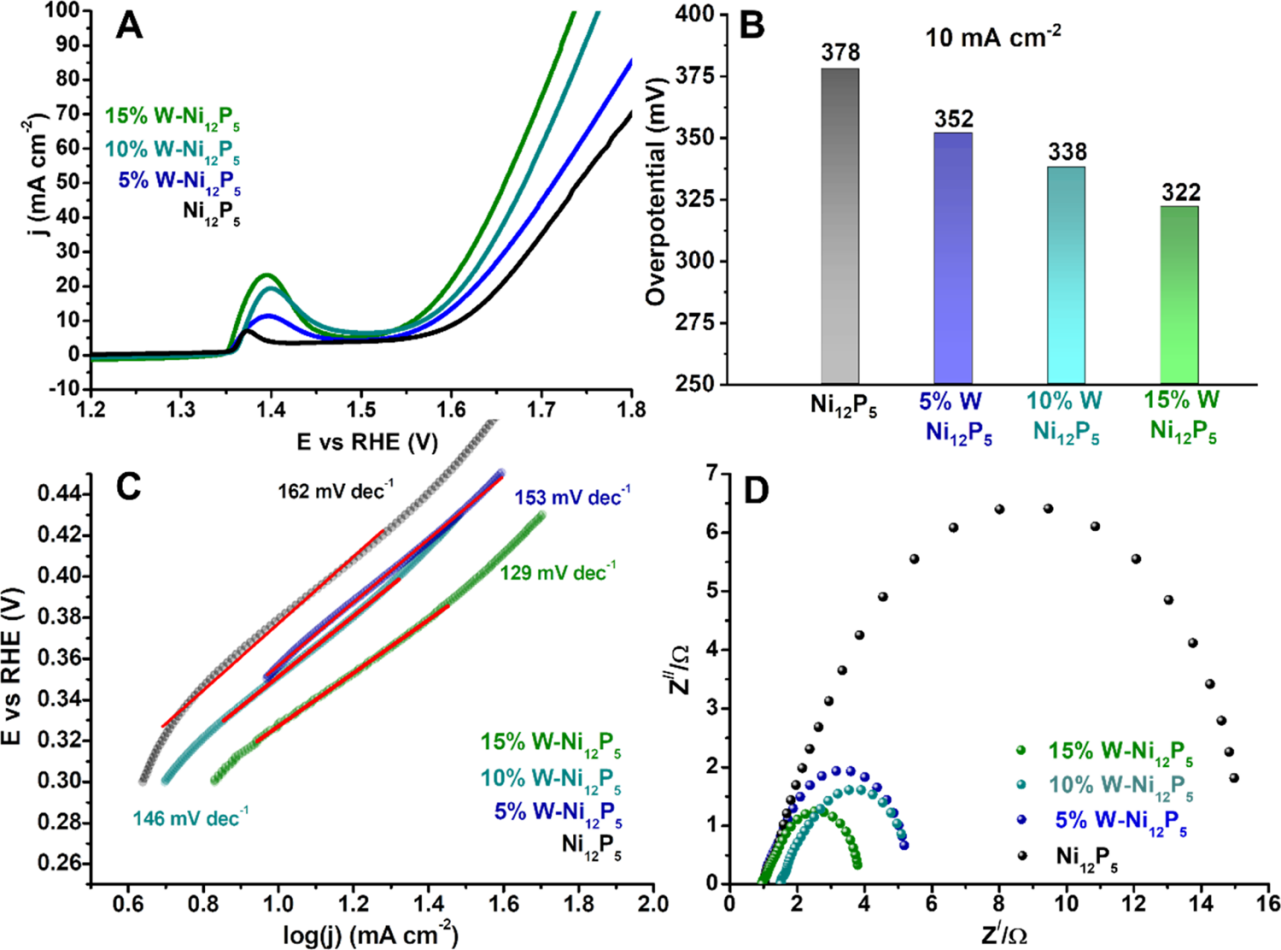
Electrochemical performances of pristine and W-doped nickel phosphide catalysts for the OER: (A) LSV polarization curves in 1.0 M KOH at a scan rate of 5 mV s^–1^, (B) overpotentials at a current density of 10 mA cm^–2^, (C) Tafel plots, and (D) Nyquist plots of the W-doped Ni_12_P_5_ samples along with pristine Ni_12_P_5_ at an overpotential of η = 340 mV for the OER.

Next, we measured the OER performance of the catalysts, still under alkaline conditions (1.0 M KOH), using a Hg/HgO electrode as the reference electrode and a graphite rod as the counter electrode. The non-iR-corrected LSV polarization curves for the OER (Figure 4A) show a trend of activity similar to HER, where W doping enhances the catalytic activity. The characteristic peak located around 1.4 V observed in all of the samples corresponds to the oxidation of Ni^2+^ to Ni^3+^, which forms the active OER species in Ni-based electrocatalysts.^29^ A slightly anodic shift of the Ni redox wave was observed upon doping, and the peak area increased with the W content within the samples (Figure 4A), which is consistent with previous reports.^30^ By integrating the wave area, we calculated the total charge transfer and confirmed that doping with W shows a continuous rise in the number of electrons transferred (Figure S10) such that 15% W-Ni_12_P_5_ transferred 6 times more electrons (and potentially formed 6 times more of NiOOH) than pristine Ni_12_P_5_, similar to the improvement of the C_dl_ values in Figure 3d. The maximal catalytic activity was observed for 15% W-Ni_12_P_5_, which exhibits an overpotential of only 322 to drive a current density of 10 mA cm^–2^ (Figure 4B) and a Tafel slope of 129 mV dec^–1^ (Figure 4C); this overpotential value is better than or comparable to that of the state-of-the-art OER catalysts in alkaline solution (see the comparison in Table S2). In addition, the charge transfer kinetics of the electrode were assessed via EIS measurements performed at η = 340 mV (Figure 4D), which show that the 15% W-Ni_12_P_5_ electrode has the lowest *R*_ct_ value (3.8 Ω), consistent with a faster charge transfer rate for the OER. Additional W content beyond 15% in the feed resulted in a phase change to Ni_3_P and poorer catalytic results (see Figure S11). We further confirmed the stability of the preferred catalyst, 15% W-Ni_12_P_5_, for the HER and OER in 1 M KOH at a constant current density of 10 mA cm^–2^ by chronopotentiometry studies (see Figure S12).

**Figure 5:**
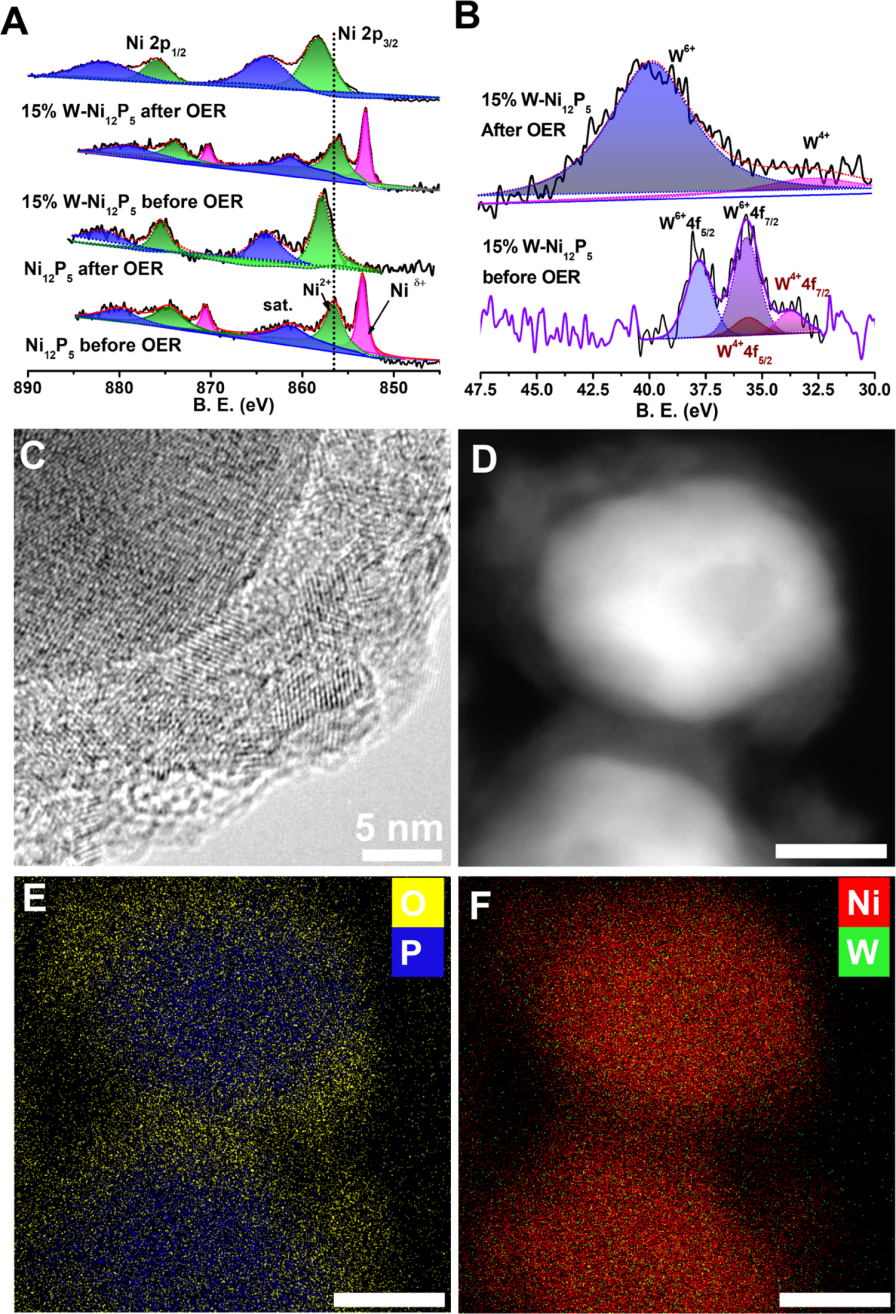
XPS spectra of (A) Ni and (B) W after 200 CV cycles of the OER. (C, D) HRTEM and STEM images of 15W-Ni_12_P_5_ after 200 CV cycles of the OER. (E, F) Element mappings of the same nanoparticle as in (D). The scale bar for (D–F) is 20 nm.

To further understand the catalytic mechanism, we characterized the catalyst electrodes before and after 200 cycles between 0.9 and 1.8 V (vs RHE). We used X-ray photoelectron spectroscopy (XPS) to investigate the surface species and detected the presence of Ni, P, W, and O (see the survey spectrum in Figure S13). Figure 5A presents the Ni XPS spectra of pristine and 15% W-doped Ni_12_P_5_ before and after the OER measurements (the XPS spectra of the full data set are available in Figure S14A,B). The signal for Ni^δ+^ (δ < 2) was attributed to Ni bonded to P within the Ni_12_P_5_ lattice, and the presence of various Ni oxide species was evidenced by signals corresponding to Ni^2+^ species. Monitoring the changes in the Ni oxidation state proved insightful. Before the catalytic experiments, Ni^δ+^ and Ni^2+^ were all present, and W doping caused a systematic shift to lower binding energies. After the catalytic experiments, the Ni^δ+^ peaks had vanished (Figure 5A), which confirms the complete oxidation of Ni_12_P_5_ to Ni–O and Ni–OOH species.^31^ We also conducted a reference experiment, where the powder catalysts were immersed in OH^–^ (1 M) an hour and then characterized (Figure S15). The XPS of the reference sample unveiled that in alkaline solution, the fraction of Ni^δ+^ was reduced from 51 to 28%, even before applying a bias. In addition, crystalline Ni(OH)_2_ is absent from the FTIR spectrum (Figure S15B), although a strong signal for OH^–^ is present, which suggests the formation of a disordered layer of oxidized Ni. The W content at the surface increased after the OER experiment, from 4.0 to 10.6% (Figure 5B). We attribute the surface enrichment with W to the harsh conditions at the surface, where P is depleted and subsequent cycles of oxidation and reduction of the Ni oxide are performed, thus offering additional mobility to the W atoms that prefer to move toward the surface. Before the catalytic experiments, phosphorus was present as phosphide in Ni_12_P_5_ and also as phosphate (Figure S14C,D).^16,32,33^ Doping with W produced a downshift in the P binding energies that is similar to the downshift noticed for Ni. The increased electron density on the phosphorus makes it a better proton acceptor, potentially promoting the cleavage of the H^δ+^–OH bond, whereas the lower positive charge on Ni^δ+^ weakens the adsorption strength toward −OH_ads_, facilitating hydroxyl desorption and preventing the poisoning of the active sites. However, after the catalytic experiments, the surface was depleted of P; in fact, the P peaks were almost undetectable in all of the samples (Figure S14D). As for W, its dominant oxidation state in the as-synthesized samples was W^6+^, but the presence of W^4+^ from surface oxidized species of WP was also detected (Figures 5B and S16).^34^ After the OER, not only W^4+^ was almost undetectable, but an upshift in the binding energies of W^6+^ was detected, which indicates the coordination of W^6+^ with the more electronegative oxygen rather than with phosphorus. This observation is also consistent with the formation of a surface layer of oxidized Ni with W doping.

Raman spectroscopy provides corroborating evidence for the effect of W doping (Figure S17): a more pronounced signal for Ni(OH)_2_ and NiO(OH) is seen for the 15% W-Ni_12_P_5_ sample after OER CV cycling,^35,36^ indicating that W doping accelerates the formation of oxidized nickel species. High-resolution transmission electron microscopy (HRTEM) of 15% W-Ni_12_P_5_ after the OER confirmed the formation of a disordered shell over the single-crystalline Ni_12_P_5_ core (Figure 5C). Scanning transmission electron microscopy (STEM) with energy-dispersive X-ray spectroscopy (EDS) element mapping of O, P, Ni, and W (Figure 5D–F) shows the presence of Ni, W, and O in the shell and Ni, W, and P in the core. Additional images are provided in the Supporting Information (Figures S18 and S19). We concluded that during the catalytic measurements, surface transformation occurs at the oxidation stages, and as a result, the (W)-Ni_12_P_5_ core is embedded in an active Ni(W)OOH layer at the external shell.

We further evaluated the OER intrinsic catalytic activity of the catalysts by calculating their turnover frequency (TOF). It was calculated as the ratio of the O_2_ evolution rate per mole of active sites at a fixed overpotential.^37^ By integrating the charge over the Ni^3+^/Ni^2+^ redox peak, we were able to calculate the density of OER active sites. The TOF was deduced from the current density plot by normalizing with respect to the surface-active sites and the geometrical surface area. Further details on the calculations of the active sites and the TOF are provided in Supporting Information Figure S20. The 15% W-Ni_12_P_5_ catalyst delivered the best TOF value: 0.09 s^–1^ at an overpotential of 350 mV. This is a better value than some of the recently reported OER catalysts, such as V-Ni_2_P (0.059 mol O_2_ s^–1^ at 300 mV),^18^ Cu(OH)_2_@CoNiCH NTs/CF (0.01739 mol O_2_ s^–1^ at 300 mV),^38^ Ni*_x_*Fe_1–*x*_OOH/NiFe/Ni*_x_*Fe_1–*x*_OOH SNTAs-CFC (0.0453 mol O_2_ s^–1^ at 350 mV),^39^ and HPGC@NiFe (0.0396 mol O_2_ s^–1^ at 300 mV).^40^

**Figure 6:**
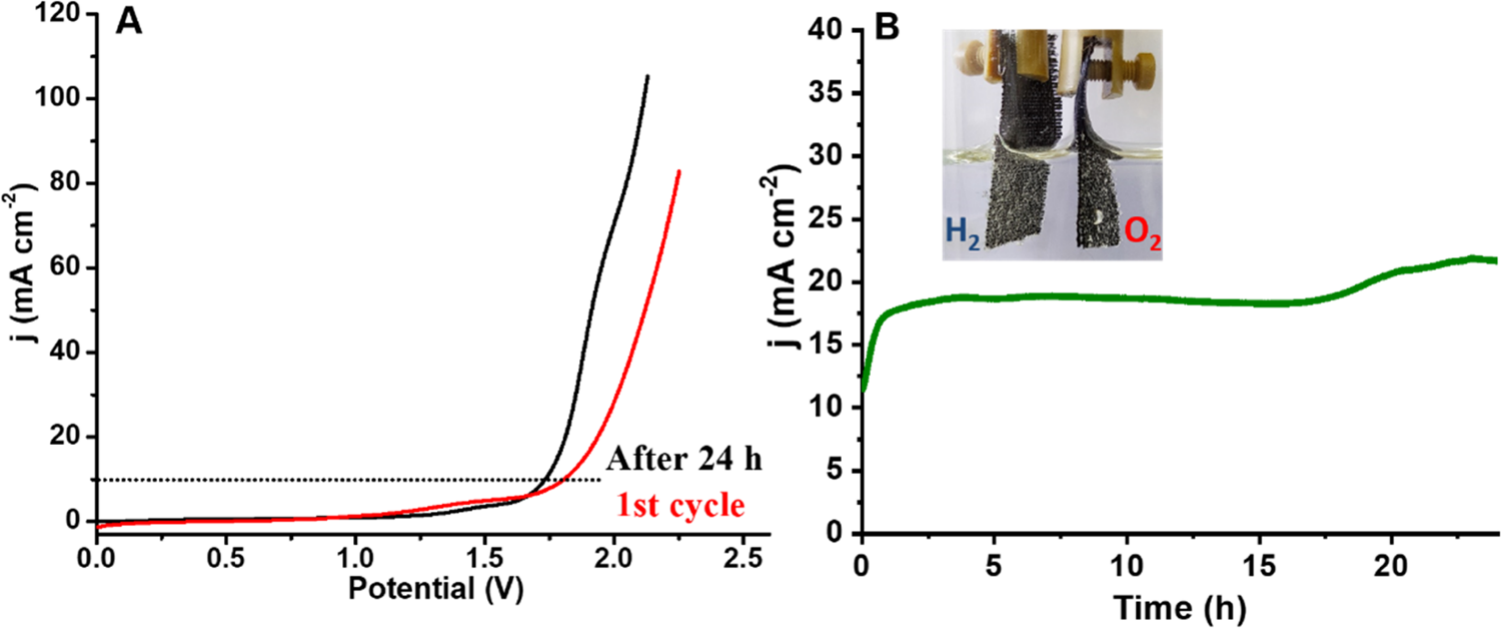
(A) Polarization curves of the 15%W-Ni_12_P_5_ electrolyzer in 1 M KOH at 5 mV s^–1^ (in a two-electrode system). (B) Chronopotentiometry curve of water electrolysis at a 2 V bias voltage in 1 M KOH; the inset shows a photograph of H_2_ and O_2_ bubbles on the surface of the electrodes during the electrolysis process.

Motivated by the bifunctionality of 15% W-Ni_12_P_5_, and to explore its prospect as a practical catalyst for overall water splitting, we assembled a two-electrode alkaline electrolyzer cell by spreading the catalyst on carbon cloth, both as the HER and OER catalysts. In this configuration, 10 mA cm^–2^ was achieved by applying only 1.8 V between the two electrodes of 15% W-Ni_12_P_5_ (Figure 6A). Moreover, in a long duration run under a cell voltage of 2.0 V (Figure 6B), the current density increased significantly during the first two hours and then stabilized, generating a considerable flow of gas bubbles in the following 20 h (see the inset in Figure 6B). After this prolonged usage, only 1.73 V was required to achieve 10 mA cm^–2^, as seen in Figure 6A (black curve), an improvement we attribute to conditioning of the OER electrode. This voltage value is similar to or better than that of several recently reported bifunctional electrolyzers (Table S3; see also the Supporting movie, which shows the working cell).

To understand the role of W in the Ni_12_P_5_, we carried out density functional theory (DFT) calculations. The computational details and the extensive preliminary calculations are available in the Supporting Information (Figures S21–S26). The main findings are summarized here as follows: W doping in Ni_12_P_5_ is endothermic irrespective of the W distribution with substitution energies of +0.4 to 0.7/W atom in both the bulk and within a thin slab that represents the nanoparticles. Therefore, when the content of W in the feed is 15%, much less is incorporated into the lattice, which is consistent with the experimental results. The W doping contributes little to the density of states (DOS) around the Fermi level, which is mostly dominated by the Ni contribution, and therefore, its effect on the electron conduction properties of the lattice is minor. In addition, the prominent W5d states are located at ca. 0.7–1.5 eV above *E*_F_; hence, they cannot participate actively in the formation of a covalent or dative bonding. The lattice parameters of γ-NiOOH are commensurate with the lattice parameters of the (101) facet of Ni_12_P_5_; this illustrates the role of the Ni_12_P_5_ platform in stabilizing this specific phase, which is otherwise less stable than the more studied β-NiOOH. The substitution of W into γ-NiOOH is still endothermic but with lower formation energies, which range from +0.12 to 0.17 eV/NiOOH, depending on the substitution site.

**Figure 7:**
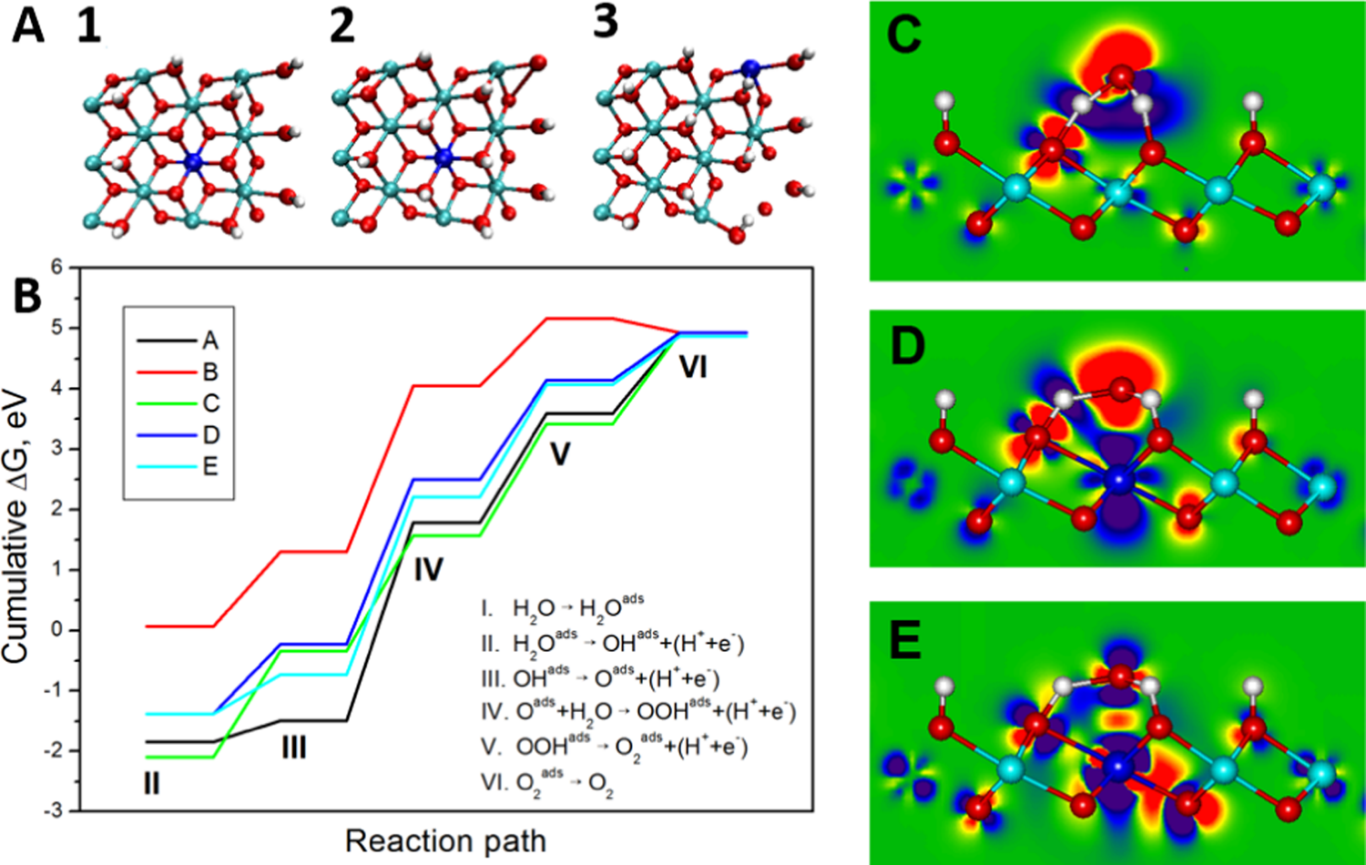
(A) Ball-and-stick models for W-doped γ-NiOOH with a single W dopant atom. Ni in cyan, W in blue, O in red, and H in white. W-doped γ-NiOOH may adopt three configurations: (1) bare WO_6_ octahedron, (2) WO_6_ with adsorbed H-atoms and three H vacancies at the NiO_6_H_3_ octahedra, or (3) a single Ni vacancy. (B) Cumulative free energies, Δ*G*, for a single-site associative reaction mechanism of the OER using γ-NiOOH doped by a single W atom. A is a pristine undoped layer; B is the structure depicted in model 1, where the bare WO_6_ octahedron serves as the reaction site; C is the structure depicted in model 2, where the WO_6_H_3_ octahedron serves as the reaction site; D is the structure depicted in model 3, where the WO_6_H_3_ octahedron serves as the reaction site; and E is the structure depicted in model 3, where the Ni vacancy serves as the reaction site. (C) Map of the electron density redistribution Δρ after the adsorption of an O atom to the NiO_3_H_3_ octahedron in pristine γ-NiOOH. (D) The same after the adsorption of an O atom to the WO_3_H_3_ octahedron in doped γ-NiOOH. (E) Difference, ΔΔρ, between (D) and (E), which shows a diminished electron density in the vicinity of the O atom adsorbed on the WO_3_H_3_ site (i.e., intermediate reaction complex at step IV in model 2).

The remaining question is what is the role of W dopants in the active layer of γ-NiOOH. Because charge neutrality in the doped samples must be maintained, the insertion of a W^6+^ ion as substitutional to Ni^3+^ must be compensated by three H vacancies or a single Ni vacancy (see models 1–3 in Figure 7A). To understand the OER activity, we calculated the change in Gibbs free energy (Δ*G_i_*) for the elementary OER reactions steps in the various γ-(Ni,W)OOH layers for a single-site associative reaction mechanism using the scheme employed earlier for β-NiOOH^41^ (see Figure 7B and Table S4). The calculations show that, in all cases, the rate-limiting step of the OER is the formation of an OOH^ads^ intermediate (step IV: *O + H_2_O → *OOH + H^+^ + e^–^, in Figure 7B). The pristine γ-NiOOH has the highest energy barrier, Δ*G*_IV_ = +3.28 eV, which is lower for the W-doped layers, reaching +2.73 to 2.94 eV and even +1.91 in the case where the WO_6_H_3_ octahedron is the reaction site (see path C in Figure 7B). For the latter, the calculated energy barrier shows an overpotential (η) of 0.68 V since η = (Δ*G*_a_ – 1.23 eV)/e, indicating a potential advantage for the OER. It should be noted that the calculated η is not fully consistent with our experimental η (at 10 mA cm^–2^), as the calculation is used here mainly to reflect the general trend.

The role of the W dopant was revealed by mapping the electron density ρ on a model of the O atom adsorbed onto the pristine or W-doped γ-NiOOH layer (Figure 7C–E), which is the starting point for reaction IV, the rate-limiting step of the OER. For a better understanding of the differences, Figure 7E shows the subtraction of the two maps, revealing that for the doped layers, the electron density is much lower in the vicinity of the adsorbed O atom and thus facilitates the attack of the nucleophilic H_2_O molecule or the OH group on the O 2p_z_ orbital of the adsorbed O atom, which leads to the formation of the desired O–O bond.

## Conclusions

In summary, we presented the W doping of Ni_12_P_5_ particles, which affords bifunctional electrocatalysts for overall water splitting. Doping with W is not favorable thermodynamically, so only small amounts can be incorporated into the lattice. The doping expands the lattice, and the outer surface of the particles with the highest doping content seems corrugated and rough, showing oxidized Ni constructs, which results in high ECSA for the electrocatalysis. The doping also considerably enhances the intrinsic HER by reducing the energy barrier for the electron-coupled water dissociation (Volmer step). For the OER, W doping promotes the formation of high-valence Ni species as a thin shell of γ-NiOOH. According to DFT calculations, the lattice parameter of Ni_12_P_5_ matches that of γ-NiOOH, which is known as the most active NiOOH phase for the OER process; this facilitates the stabilization of this otherwise poorly stable phase and results in a structure with Ni and H deficiencies. In this configuration, the W dopants hardly contribute to the density of states (DOS) around *E*_f_ and do not directly participate in the electrochemical process. Their main role is to reduce the electron density on the adsorbed *O atom in the rate-determining step of the OER and thus facilitate oxygen evolution. When utilized as both the anode and the cathode, the 15% W-Ni_12_P_5_ catalyst affords an overall water splitting current density of 10 mA cm^–2^ at a cell voltage of only 1.73 V with good stability, which allows good prospects in practical water electrolysis.

## Supporting Information Available

FTIR, EDS element mapping, ICP-OES, XPS and Raman spectra, and details of the DFT calculations (PDF)
Movie of the electrolyzer cell during operation (MP4)
