# Supplementary material for: W Doping in Ni12P5 as a Platform to Enhance Overall Electrochemical Water Splitting
Source: ACS Appl Mater Interfaces. 2021 Dec 28;14(1):581–9. doi: 10.1021/acsami.1c16755 (PMC8762645; doi:10.1021/acsami.1c16755)
Supplement: Supplementary file 1 — am1c16755_si_001.pdf [file am1c16755_si_001.pdf]

Supporting information

W doping in $Ni_{12}P_5$ as a platform to enhance overall electrochemical water splitting

*Sirshendu Ghosh[1], Sunil R. Kadam[1], ShayLee Kolatkar[1], Alevtina Neyman[1], Chanderpratap Singh[1], Andrey N. Enyashin,[2,3] Ronen Bar-Ziv,[4] and Maya Bar-Sadan[1]**

[1]Department of Chemistry, Ben-Gurion University, Beer-Sheva, 8410501, Israel

[2] Institute of Solid-State Chemistry UB RAS, 620990 Ekaterinburg, Russian Federation

[3] Institute of Natural Sciences and Mathematics, Ural Federal University, 620075 Ekaterinburg, Russian Federation

[4] Chemistry Department, Nuclear Research Centre−Negev, P.O. Box 9001, Beer-Sheva 84190, Israel

*barsadan@bgu.ac.il

Materials:

Nickel acetylacetonate (Ni(acac)$_2$, 99.9%, Acros organics), tungsten hexachloride (WCl_6, ›99.9%, Aldrich), Oleylamine (OLAM, 70%, tech), octadecene (ODE), trioctylphosphine (TOP, 97%), hexafluorophosphoric acid (HPF_6, 65 wt%), Formamide from Sigma-Aldrich. All chemicals were used as received without any purification. Solvents used for storage and washing were purchased from Gadot and Romical. Water used for cleaning the electrode and electrochemical measurement was with resistance 18.2 MΩ cm.

Electrocatalytic measurements for water splitting:

Polarization curves were recorded on an Ivium Technologies Vertex potentiostat/galvanostat(V74606) and analyzed using the IviumSoft program. The electrolyte was bubbled with Ar gas for 15 min prior to the measurements in order to remove dissolved gases from the solution. For HER activity study, three electrode set up containing carbon rod and Ag/AgCl (in KCl) as counter and reference electrode was used. Each electrode was pre-treated with cyclic voltammetry (CV) cycles between 0 V and -0.5V (vs. RHE, reversible hydrogen electrode) until a steady voltammogram curve was obtained (usually 15 cycles).

Polarization curves were recorded at a scan rate of 10 mV s^{-1} over the same potential range. During the electrochemical measurements, the reactor headspace was continuously purged with Ar gas. All measurements were referred to the RHE using the relationship: E(RHE) = E(Ag/AgCl) + E^0(Ag/AgCl) + 0.059V × pH.

Rct values were determined by fitting the obtained semicircle with R (RC) circuit.[1]

For the OER experiment, Hg/HgO (1 M NaOH) reference electrode was used along with carbon rod as counter electrode. 200 CV cycles (at 100 mVs^{-1} in potential window of 0.8 to 1.6 V (vs. RHE)) were run for the activation of catalyst prior to LSV measurement. Polarization curves were recorded at a scan rate of 10 mV s^{-1} over 0.8 to 1.9 V (vs. RHE).

Materials Characterizations

XRD: Panalytical Empyrean powder X-ray diffractometer equipped with a position-sensitive X'Celerator detector using Cu Kα radiation (λ = 1.5405 Å) operated at 40 kV and 30 mA.

TEM: High resolution transmission electron microscopy (HRTEM) imaging was carried out using a JEOL JEM-2100F analytical TEM operating at 200 keV equipped with GATAN 894 US900 camera. Energy-Dispersive X-ray Spectroscopy (EDS) analysis was performed using a JEOL JEM-2100F TEM operating at 200 kV equipped with a JED-2300T energy dispersive X-ray spectrometer. Scanning TEM (STEM) images were taken using an GATAN 806 HAADF STEM detector. The probe size during the analysis was set to 1 nm. JEOL Analytical Station software (v. 3.8.0.21) was used for the EDS data analysis. The quantitative analysis was performed by the standard less Cliff−Lorimer method.

SEM: The surface morphologies of the nanoparticles were investigated by JEOL JSM-7400F ultrahigh resolution cold FEG-SEM. The sample for SEM was prepared by drop-casting the nanoparticles dispersion in $CHCl_3$ on a silicon wafer.

XPS: X-ray photoelectron spectrometer ESCALAB 250 ultrahigh vacuum (1 × 10^{-9} bar) apparatus with an Al Kα X-ray source and a monochromator.

Raman: Room temperature Raman spectroscopy were performed using a LabRAM HR Evolution Raman spectrometer with a laser wavelength of 532 nm in the back-scattering geometry. The laser power on the sample was 0.6 mW with a laser spot size of 1.3 µm. An inductively coupled plasma optical emission spectrometer (ICP-OES) from Spectro Arcos

which was calibrated with certified standards. For ICP-OES measurements, the powder Ni-P material was dissolved in aqua regia (with few drops of hydrogen peroxide) and was heated at 70 °C for 2 hours, to decompose the solid compound into its components.

FTIR: Infrared (FTIR) spectra were acquired from KBr pellets using a Nicolet Impact 410 spectrophotometer.

Ligand stripping

Procedure: To make the nanocrystals surface more catalytically active, we replaced the long organic chained OLAM and TOP with PF_6^- ligand. Dried powder of the as-synthesized nanocrystals (10 mg) was dissolved in 2 ml $CHCl_3$ and mixed with equal volume of HPF_6 solution (in formamide). The mixture was agitated in a vortex genie for 2 min. Within 1 min, the ligand exchanged nanocrystals were flocculated. The mixture was centrifuged at 6000 rpm for 5 min and the product re-dissolved in 1 ml $CHCl_3$ and the same volume of HPF_6 solution. The solution was agitated for 2 min for complete removal of the organic ligands and centrifuged. The product was re-dissolved in 2 ml of $CHCl_3$ and 2 ml of acetone and sonicated mildly for 3 min, and then the nanocrystals were collected by centrifugation. The washing process was repeated two times for a complete removal of OLAM, TOP and unreacted HPF_6. The collected product was vacuum-dried and stored in a glovebox.

FTIR Analysis:

Figure S1 shows the FTIR spectra of OLAM, TOP and as-synthesized $Ni_{12}P_5$. For the as-synthesized $Ni_{12}P_5$ sample, the stretching vibration of the -C-H- group of OLAM at 2854.5 cm^{-1} and TOP at 2921.28 cm^{-1} and the bending vibration of -C-N- and –N-H- of OLAM at 1060 cm^{-1} and 1555 cm^{-1} were identified in the spectrum. This observation along with the shifting and broadening of the bands confirmed the binding of OLAM onto the nanocrystals’ surface. For ligand exchanged product, the absence of the stretching modes of -C-N-, –N-H- and –C=C- confirmed the complete elimination of OLAM and TOP from the nanocrystals’ surface.

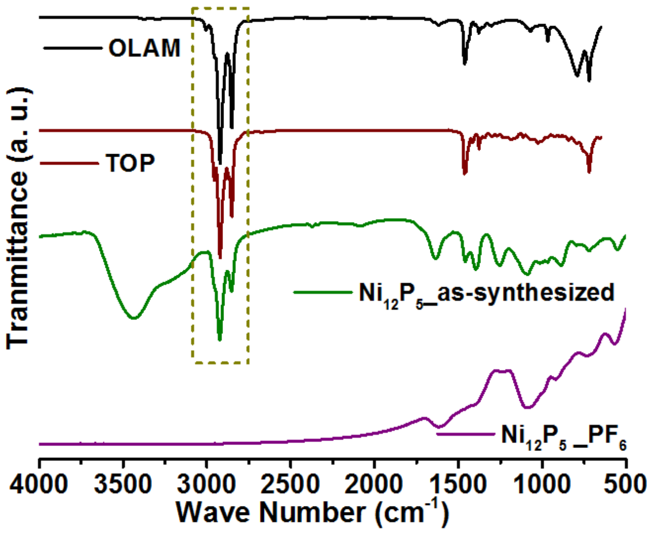

Figure S1. FTIR spectra of as-synthesized $Ni_{12}P_5$, OLAM and TOP and the PF_6^- ligand-exchanged $Ni_{12}P_5$.

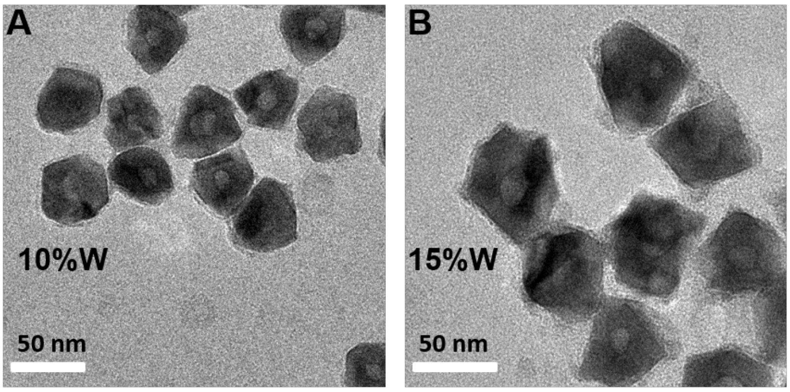

Figure S2. TEM images an assortment of (a) 10%W and (b) 15%W doped $Ni_{12}P_5$ nanoparticles.

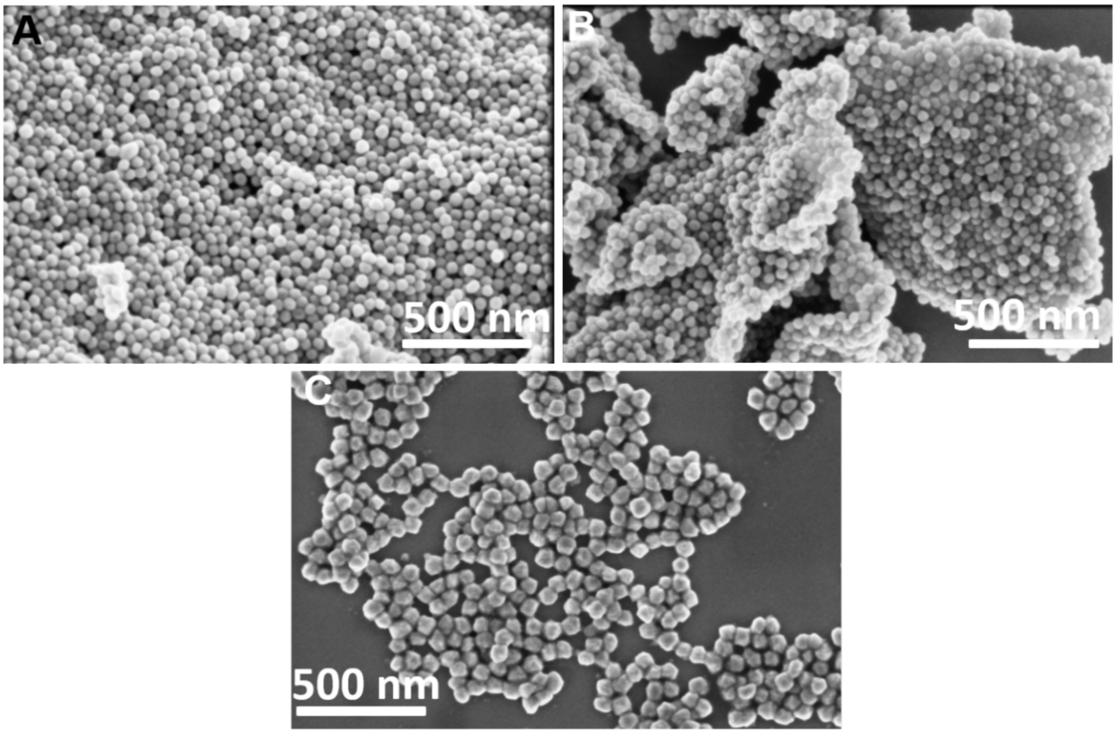

Figure S3. SEM images of (A) 5%W, (B) 10%W and (C) 15% W doped $Ni_{12}P_5$.

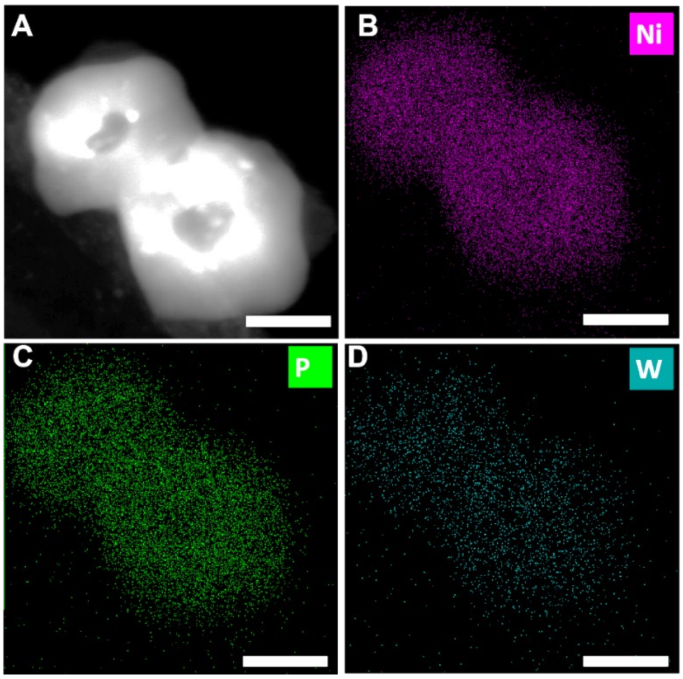

Figure S4. (A) HAADF-STEM image of 15 %W-$Ni_{12}P_5$ nanoparticles. (B–D) element mapping for Ni, P and W, respectively. Scale bar is 25 nm.

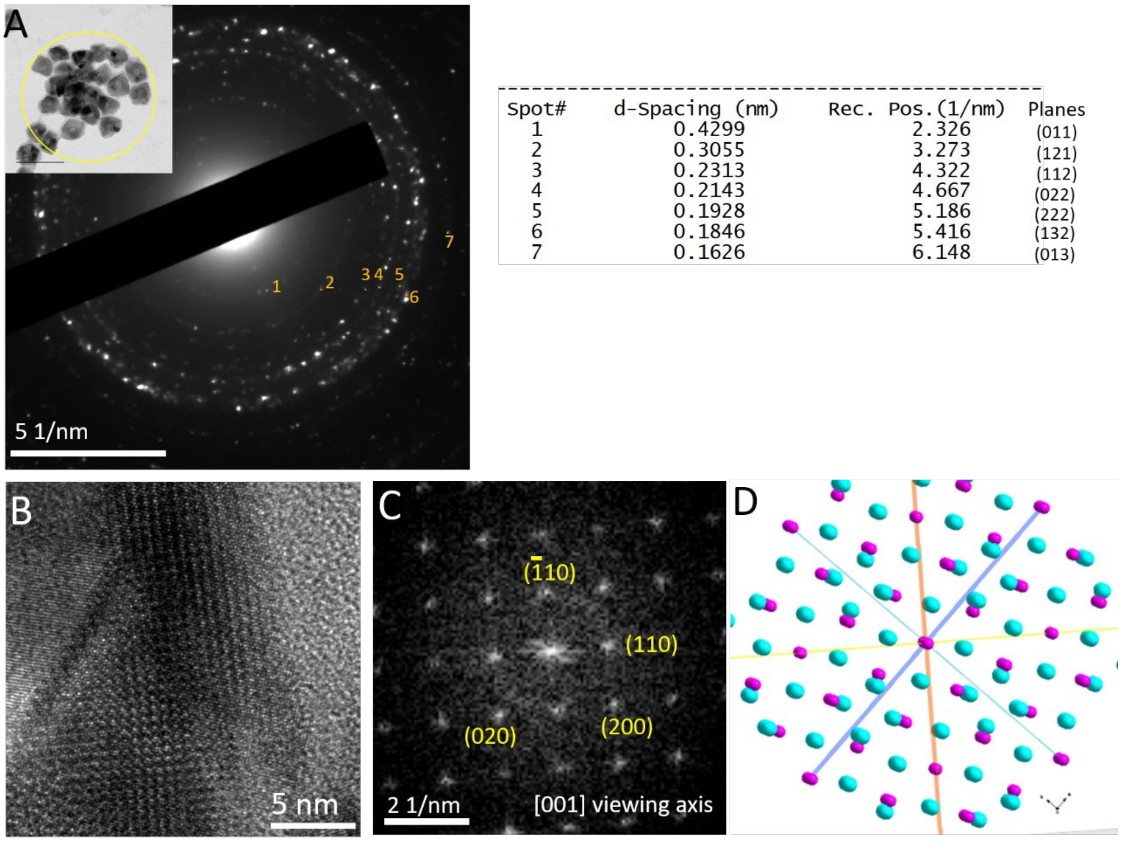

Spot#	d-Spacing (nm)	Rec. Pos.(1/nm)	Planes
1	0.4299	2.326	(011)
2	0.3055	3.273	(121)
3	0.2313	4.322	(112)
4	0.2143	4.667	(022)
5	0.1928	5.186	(222)
6	0.1846	5.416	(132)
7	0.1626	6.148	(013)

Figure S5. (A) Selected area electron diffraction (SAED) of as-prepared 15%W-$Ni_{12}P_5$ nanoparticles. Inset shows the area from which the SAED was taken. Right panel shows the major indexed planes and corresponding d-spacing value calculated from the SAED pattern. (B) HRTEM image and (C) corresponding FFT pattern of 15%W-$Ni_{12}P_5$ nanoparticle. (D) Atomic model of $Ni_{12}P_5$ with assigned planes from FFT pattern along the [001] zone axis.

Table S1. Ratio of $Ni^{\delta+}$ and Ni^{2+}, W concentration in different as-synthesized nickel phosphide nanocrystals:

	$Ni_{12}P_5$	5% W-$Ni_{12}P_5$	10% W-$Ni_{12}P_5$	15% W-$Ni_{12}P_5$
**$Ni^{2+/3+}$/ $Ni^{\delta+}$**	0.67	0.81	1.01	0.80
**PO/$P^{\delta-}$(Ni-P)**	0.87	1.12	0.90	1.01
**W% (atomic) (ICP-OES)**	--	0.6	1.7	1.7
**W% (atomic) (XPS analysis)**	--	2.3	2.9	4.0
**Unit cell volume expansion relative to $Ni_{12}P_5$ (%)***	--	0.6	2.4	2.5

* Unit cell volume expansion (in percentage) was calculated using the formula:

% unit volume expansion = $\frac{V_{doped\,Ni_{12}P_5} - V_{Ni_{12}P_5}}{V_{Ni_{12}P_5}}$ x 100

The unit cell volume for each sample was calculated using:

V = a^2c

(where *a,b,c* are the lattice parameters and a=b).

The lattice parameters *a* and *c* were calculated using the equation:

The d-spacing was calculated using the Bragg equation:

$$d_{hkl} = \frac{n\lambda}{2sin\theta_{hkl}}$$

n= 1, λ= 0.154 nm, θ_{hkl}= value taken from the XRD peak position.

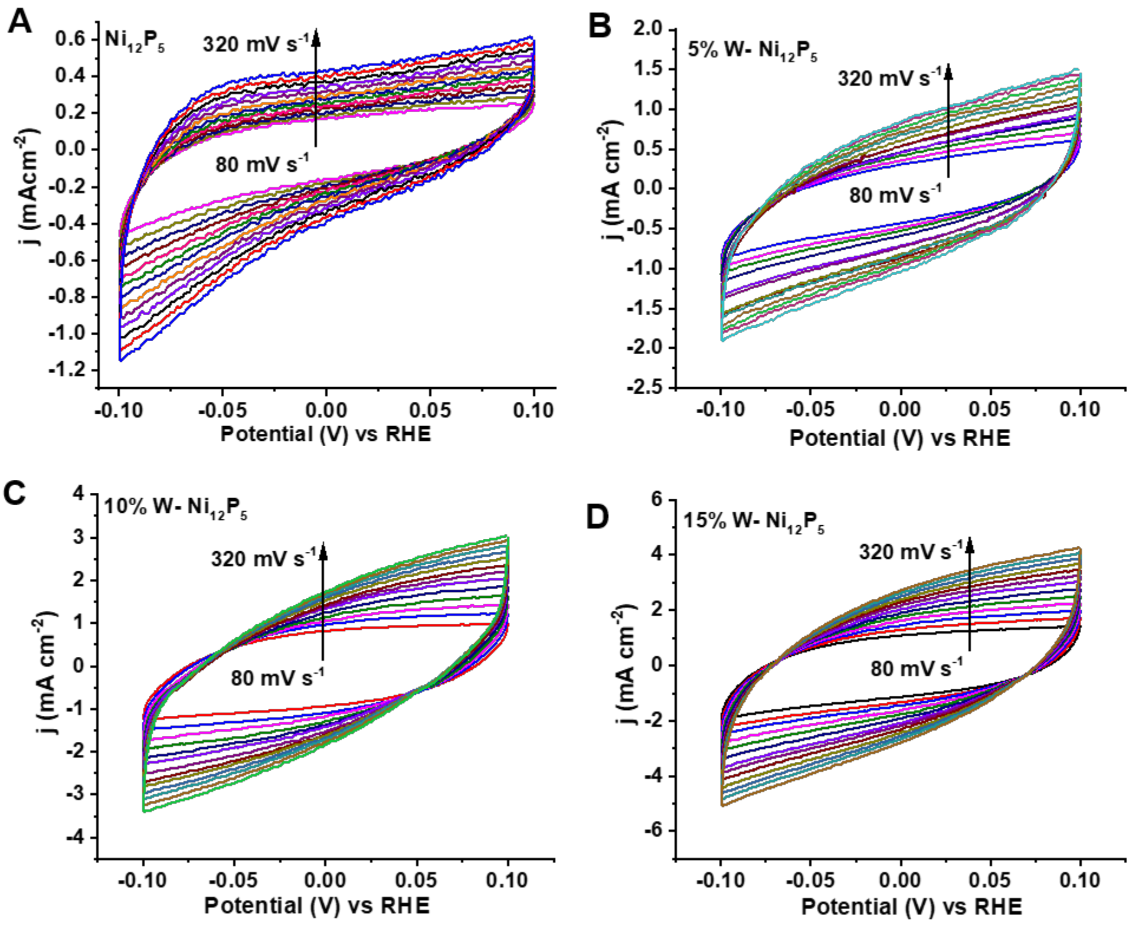

Figure S6. Cyclic Voltammetry curves at different scan rates in a non-Faradaic region for (A) pure $Ni_{12}P_5$, (B) 5% W, (C) 10% W, (D) 15% W.

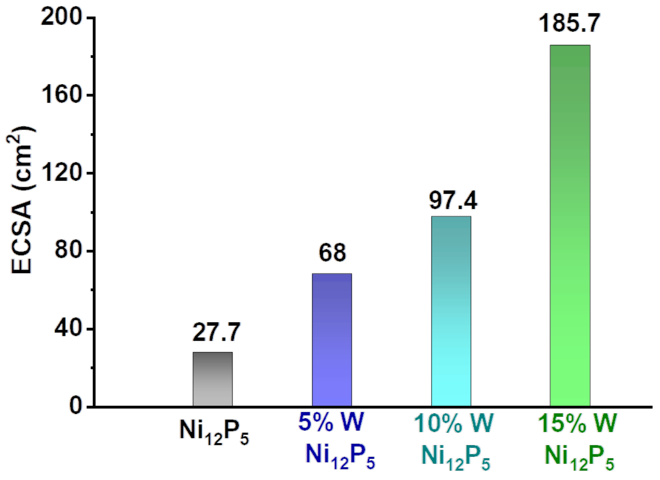

Figure S7. Comparative plots of ECSA values of different catalysts.

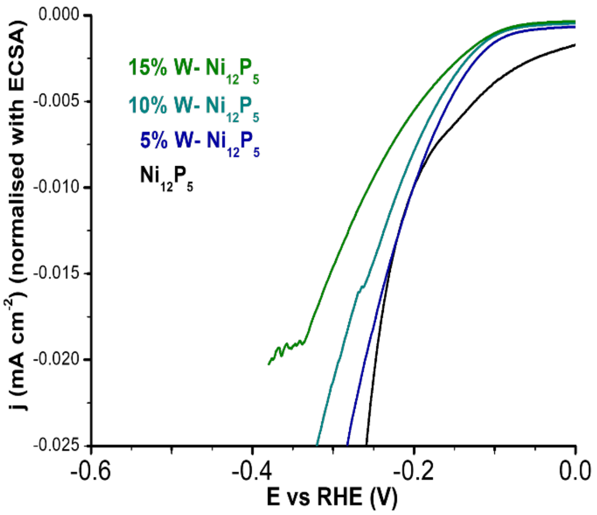

Figure S8. LSV curve for all the catalysts, normalized by the ECSA.

Table S2. Comparison of the electrocatalytic OER performance of various metal phosphide-based catalysts

Catalyst	Overpotential (mV)	References
**15%W doped $Ni_{12}P_5$ nanosphere**	**322**	**Our Work**
Co_3O_4/Ni foam	356	2
$NiS_2/CoS_2/C$	310	3
NiCoFeP/C nanosphere	270	4
CoO/CoS_x nanosphere	303	5
Mo-doped Ni_2P hollow Cubes	270	6
$Au/Ni_{12}P_5$ core/shell	340	7
Co/CoP	340	8
PdNiP-nanoparticle	330	9
O doped CoP	310	10

CNT-$Co_{1.30}Ni_{0.70}P_x$	338.2	11
CNT-$Co_{1.25}Mn_{0.75}P_x$	362.5	
CNT-$Co_{1.25}Cu_{0.75}P_x$	369.0	
CoP-Ni_2P	320	12

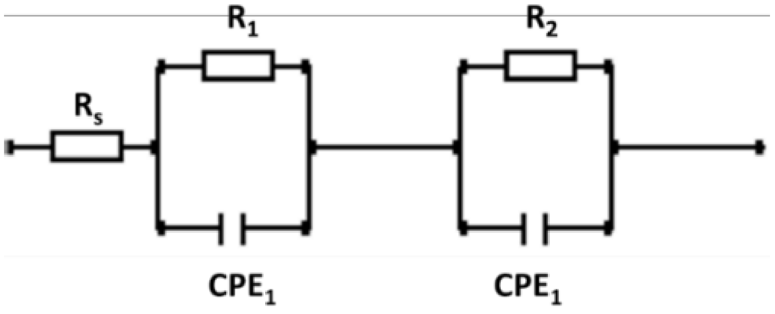

Figure S9. Equivalent circuit used to model the EIS data, where Rs represents the solution resistance, R_1 represent the charge transfer resistance and R_2 represent contact between the electrode and catalyst layer.

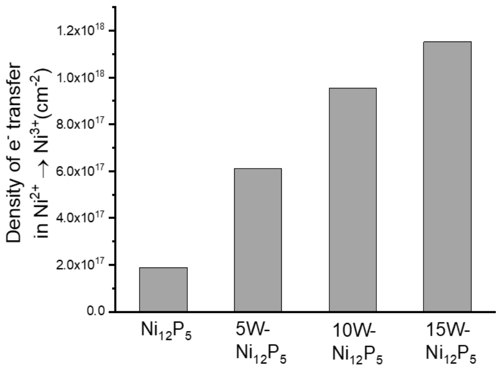

Figure S10. The total number of electrons per cm^2 transferred by the $Ni^{2+}$to Ni^{3+} conversion during the OER process. Calculated by integrating the wave area around 1.37-1.40 V (vs RHE) in **Figure 4A**, according to ref [13].

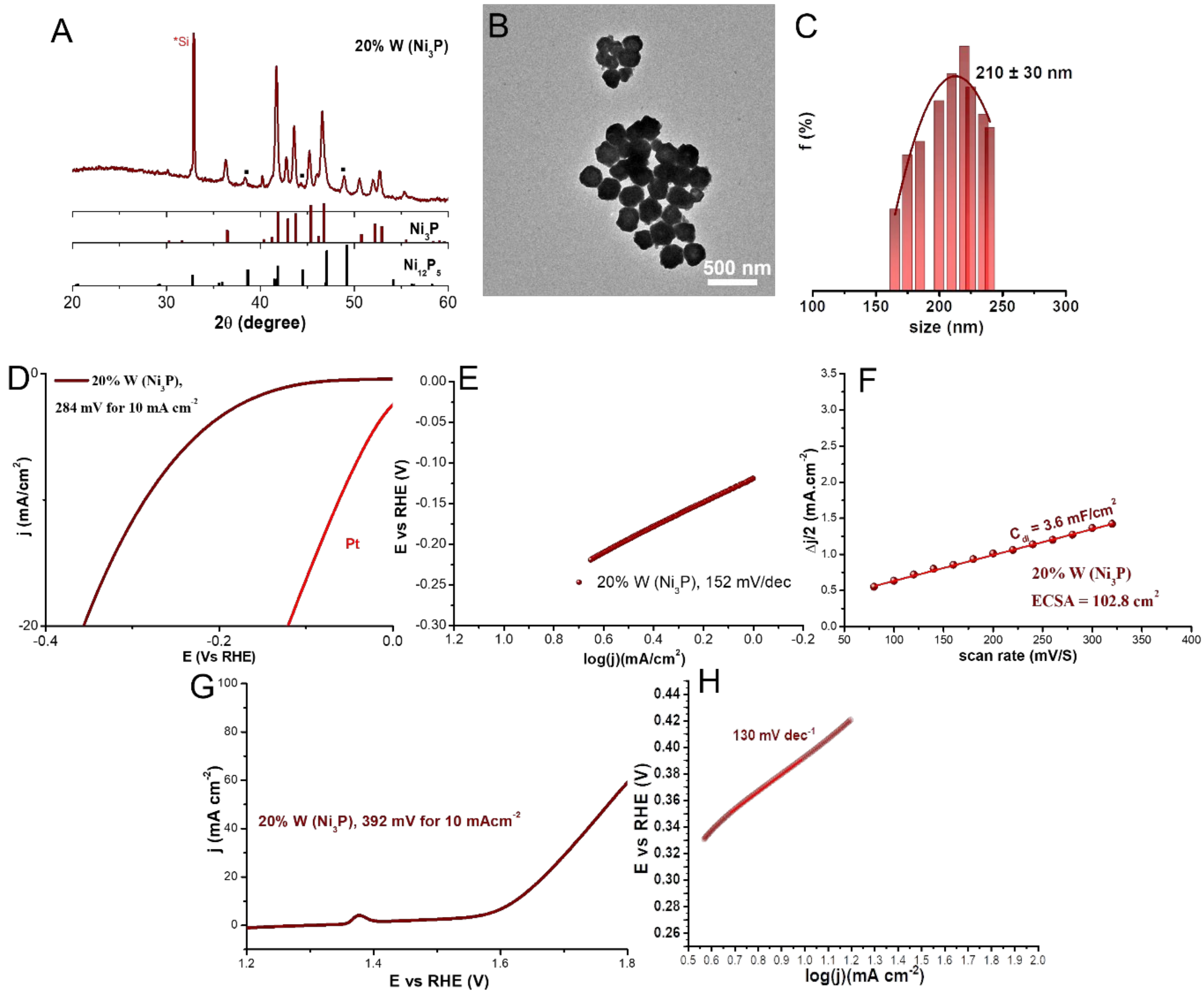

Figure S11. Summary of the characterization of 20% W-doped $Ni_{12}P_5$. (A) XRD pattern, (B-C) TEM image and size distribution. (D-F) LSV for HER in 1 M KOH, with the corresponding Tafel plot and ECSA. (G-H) LSV for OER and its corresponding Tafel plot.

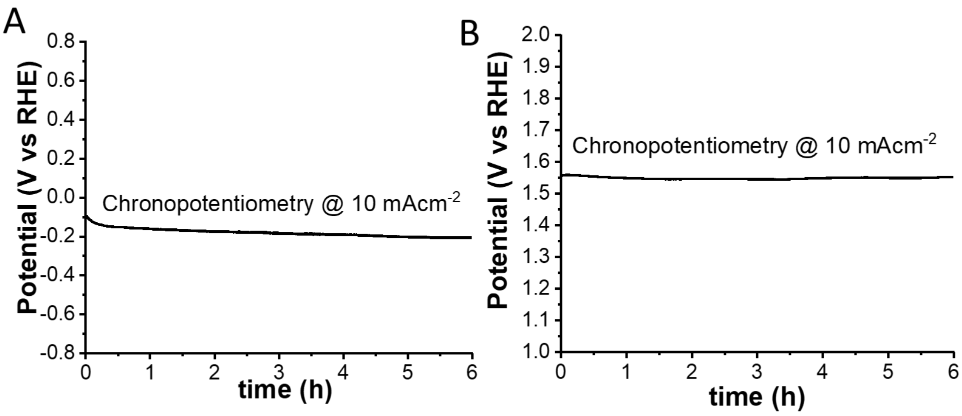

Figure S12. Chronopotentiometry of 15% W-doped $Ni_{12}P_5$ at a constant current density of 10 mA cm^{-2} in 1 M KOH for (A) HER and (B) OER.

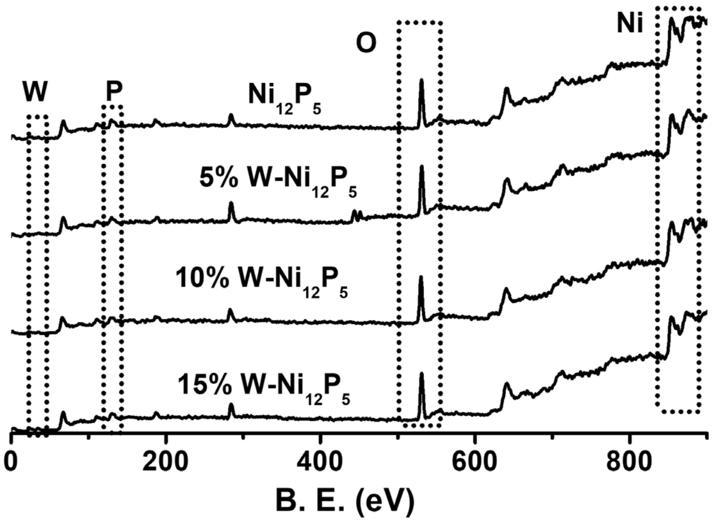

Figure S13. Survey X-ray photoelectron spectra of the as-synthesized catalysts

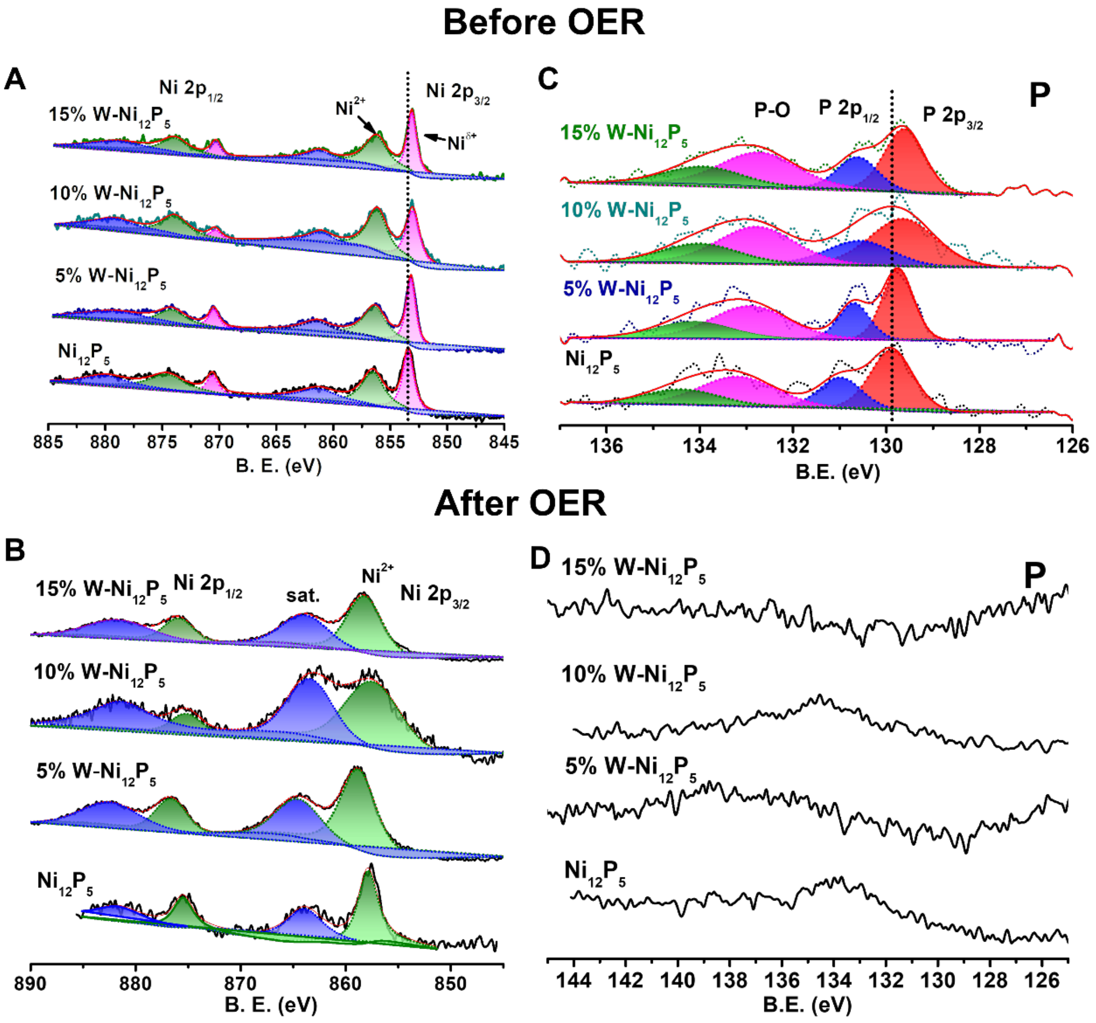

Figure S14. XPS spectra of pristine and W-doped $Ni_{12}P_5$ catalysts before and after OER measurements. Ni (A,B) and P (C,D).

To understand the effect of alkaline environment on the catalysts, we dispersed them in 1 M NaOH for 1 hr. They were collected and washed with water and ethanol two times and dried for further characterization (**Figure S15**). The XPS spectra of the treated nanoparticles confirmed an increase in the presence of oxidized Ni species, demonstrated on 5% W-$Ni_{12}P_5$: from 49% of Ni^{2+} in the as-synthesized sample to 72% Ni^{2+} after NaOH treatment. The FTIR spectra of the treated nanoparticles show the presence of surface absorbed -OH moieties, seen at 3432 cm^{-1} and the bending of absorbed water molecules observed at 1632 cm^{-1}. The characteristic feature of Ni–O–H bending and Ni–O stretching vibrations of crystalline $Ni(OH)_2$ at 520 cm^{-1} were absent.

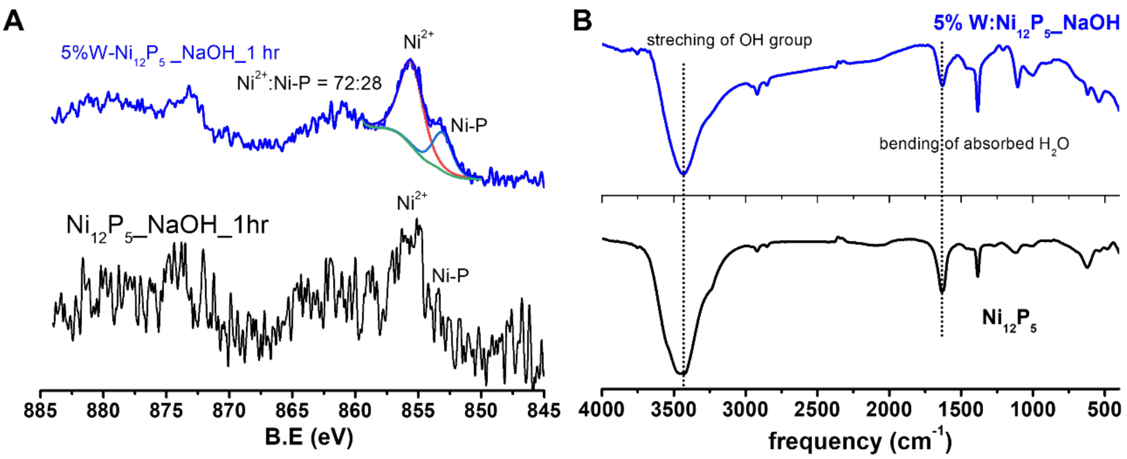

Figure S15. (A) XPS and (B) FTIR spectra of $Ni_{12}P_5$ and 5% W-$Ni_{12}P_5$ catalysts after treatment with 1 M NaOH for 1 hr.

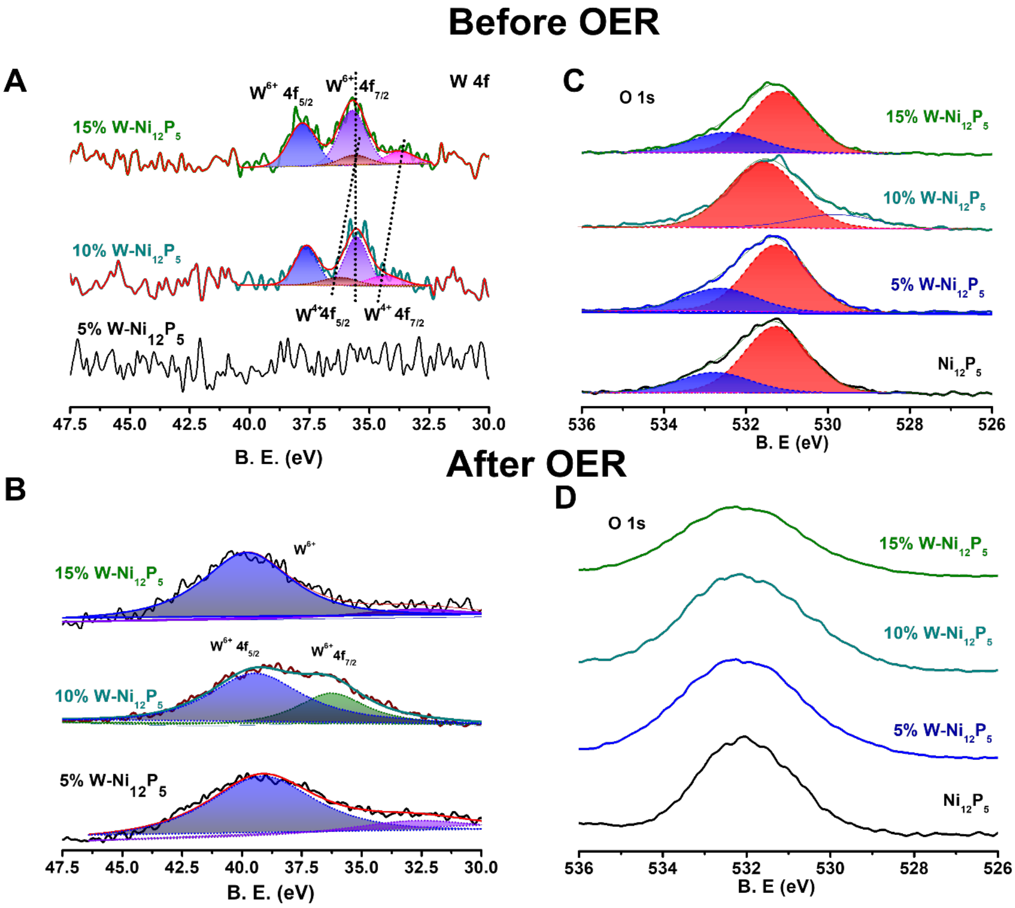

Figure S16. XPS spectra of pristine and W-doped $Ni_{12}P_5$ catalysts before and after OER measurements. W (A,B) and O (C,D).

The Raman spectra of the catalysts after OER show a prominent peak of the Ni-O bending vibration (? ?$_{Ni^{III}-O}$) at 480 cm^{-1} and a stretching vibration mode ($\upsilon_{Ni^{III}-O}$) at 562 cm^{-1}. The I_{480}/I_{562} peak intensity ratio was 1.9 and 1.78 for 15% W-doped $Ni_{12}P_5$ and pristine $Ni_{12}P_5$ respectively. This is a characteristic intensity ratio for the formation of γ-NiOOH.[14-17] The stretching vibration of Ni-O bond was also noticed at 520 cm^{-1} for $Ni(OH)_2$.

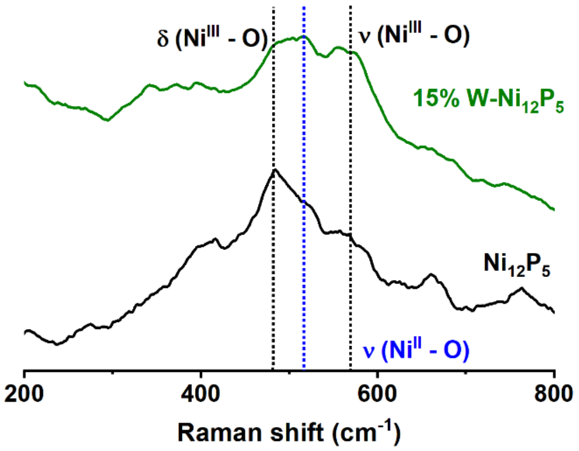

Figure S17. Raman spectra of pristine and 15%W doped $Ni_{12}P_5$ after OER (200 CV cycles in the range 0.9 V to 1.8 V (vs RHE).

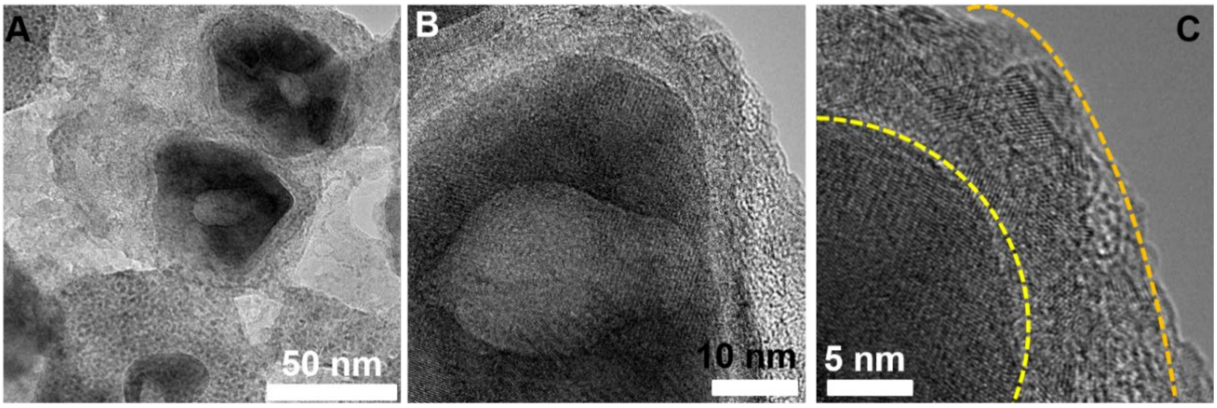

Figure S18. Electron microscopy of 15%W-$Ni_{12}P_5$ after OER (200 CV cycles) showing the formation of polycrystalline shells. (A-B) TEM (C) HRTEM.

The HAADF image shows the formation of core-shell morphology. Element mapping shows the presence of W both in core and shell region of the structures. Overlapping maps of O and P (**Figure S19F**) shows the depletion of the P from the external layer and the oxidized surface.

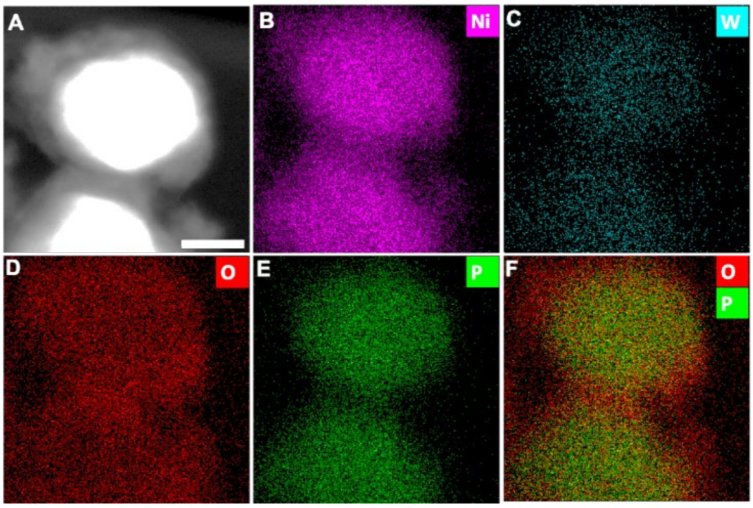

Figure S19. (A) HAADF-STEM image and element mapping of 15% W-$Ni_{12}P_5$ after OER experiments (200 CV cycles). Scale bar is 25 nm.

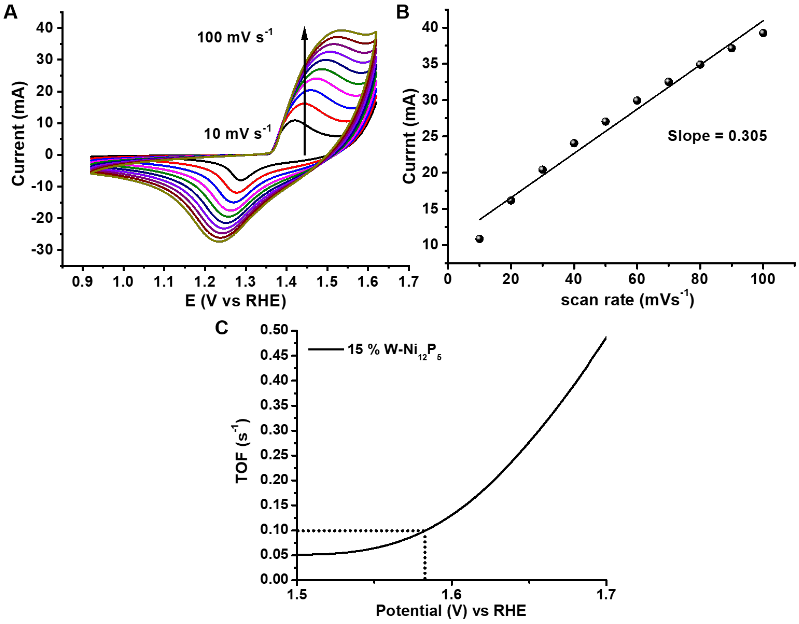

Figure S20. Cyclic voltammograms curves of (A) 15%W-$Ni_{12}P_5$ at different scan rates from 10 to 100 mV s^{-1}. (B) Variation of the peak current for oxidation with the scan rate follows linear relationship. (C) Plot of TOF for 15% W-$Ni_{12}P_5$ as a function of overpotential.

Turnover frequency (TOF) calculation:

The TOF was calculated from **Figure S20A**. From the linear relationship between the oxidation current of the redox species (Ni^{2+} to Ni^{3+}) and the scan rates (**Figure S20B**), the slope obtained is 0.305. The quantity of active species (c) was calculated from the slope using the following equation:

$$\text{Slope}=\frac{n^2F^2c}{4RT}$$

Where n, the number of electrons transferred (n=1), F represents Faradic constant, c is the number of active species, R and T are the ideal gas constant and absolute temperature (298 K), respectively. TOF values are calculated by the formula:

$$\text{TOF} = \frac{j}{4Fc}$$

J is the LSV-current density (A cm^{-2}) at specific overpotential, 4 represents the consumed electrons per catalytic site (4 for OER) and C represents the density of active sites (mol cm^{-2}).

Table S3. Comparison of the electrocatalytic overall water splitting in 1 M KOH of various catalysts

Catalyst	Overall Water splitting (V) (at 10 $mAcm^{-2}$)	References
**15%W doped $Ni_{12}P_5$ nanosphere**	**1.73**	**Our Work**
Fe_3C-Co/NC	1.77	18
CoP(MoP)-$CoMoO_3$@CN	1.724	19
FeCo-FeCoNi	1.687	20
Ni_5P_4/ Ni_5P_4	1.7	14
$NiCo_2O_4$	1.65	21

Density functional theory calculations

Computational Details

The density-functional theory (DFT) spin-polarized calculations were performed using the SIESTA 4.0 package.[22] Exchange-correlation potential was described within Generalized Gradient Approximation (GGA) in the Perdew-Burke-Ernzerhof (PBE) parametrization. The core electrons were treated within the frozen core approximation, applying norm-conserving Troullier–Martins pseudopotentials.[23] The valence shells were taken as $5d^5 6s^1 6p^0$ for W, $3d^8 4s^2 4p^0$ for Ni, $2s^2 2p^4$ for O and $1s^1$ for H. The single-ζ basis set was used for description of valence orbitals. The *k*-point mesh was generated by the method of Monkhorst and Pack with a cutoff of 15 Å for *k*-point sampling.[23] The real-space grid used for the numerical integrations was generated with the energy cutoff of 300 Ry. The calculations were performed using both variable-cell and atomic position relaxations with convergence criteria of the maximum residual stress of 0.1 GPa for each component of the stress tensor and the maximum residual force component of 0.05 eV/Å. A vacuum space of 20 Å was installed along the out-of-plane direction of the layers of the slabs (or along all three directions for individual molecules) to eliminate spurious interactions between their periodic images.

The optimized lattice parameters found using our computational scheme for the bulk phases of $Ni_{12}P_5$, β-NiOOH and γ-NiOOH are in a fair agreement with the experimental data, namely: $a = b = 8.65$ Å, $c = 5.11$ Å (for $Ni_{12}P_5$); $a = 2.95$ Å, $b = 5.14$ Å, $c = 8.79$ Å (for β-NiOOH with 2 layers per unit cell and 50%/50% H-population of their sides); $a = 2.97$ Å, $b = 5.12$ Å, $c = 8.07$ Å (for γ-NiOOH with 2 layers per unit cell and 0%/100% H-population of their sides).

Results

Thermodynamic stability and electronic properties of W-doped $Ni_{12}P_5$

The literature provides theoretical calculations regarding the most thermodynamically stable surfaces of $Ni_{12}P_5$, showing a multitude of variants such as (001), (100), (110), (101) and (111)[24] or (222), (-211) and (240).[25-27] Such an abundance of surface types suggests a negligible difference in their stabilities and reactivities within a real environment. Since we know from the experimental evidence that in the alkaline medium the external layer of the as-prepared particles is transformed to NiOOH, we focused on the (101) facet of $Ni_{12}P_5$ which shows only a 2% mismatch with a NiOOH layer. Moreover, the lattice of $Ni_{12}P_5$ along

the [101] direction can be intuitively cleaved into the non-polar Ni_2P and Ni_3P slices, which combination gives nearly the same atom density per surface area as in the NiOOH layer and demonstrates an irregular hexagonal packing of Ni atoms (**Figure S21**). From here, we employed as the model substrates the unit cells of (101) slabs with Ni_3P-Ni_2P-Ni_3P stacking and compositions $Ni_{36}P_{14}$ (=$Ni_{12.86}P_5$) or $Ni_{32}W_4P_{14}$.

The DFT data show that $Ni_{12}P_5$ can host W either as a single atom or as a cluster. While the actual doping levels in this study are lower than 2%, we calculated for the case of 4% W doping and a slight increase of the lattice parameters was evident (a = 8.69 Å, c = 5.16 Å). The calculations confirm a volume expansion upon W doping of the $Ni_{12}P_5$ lattice: 2.2% for the substitution by 4.2 % Ni atoms. Irrespective of the W distribution, the formation of $(Ni,W)_{12}P_5$ solid solutions was found as endothermic, compared with equimolar mixtures of $Ni_{12}P_5$ and a hypothetical $W_{12}P_5$. The substitution energies are +0.4 to 0.7 eV/W-atom, depending on the doping level, the substitution site and whether it is single atoms or a cluster. Therefore, W-doping of $Ni_{12}P_5$ is not favorable, thus explaining the low doping levels even when the W content in the feed was 15%.

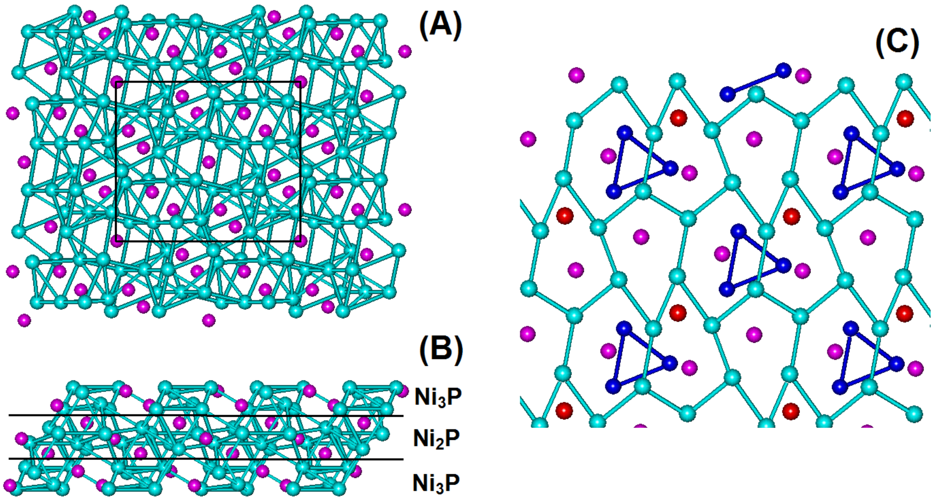

Figure S21. Ball-and-stick model of a $(101)Ni_{12}P_5$ slab (Ni in cyan and P in violet): (A) Top view with the unit cell framed; (B) Side view with the selected stoichiometric non-polar "slices"; (C) Top view on a single Ni_2P-slice (Ni in cyan and P in violet) and a Ni_3P-plane underneath (Ni in blue and P in red) uncovering an irregular hexagonal network of Ni-atoms with nearly the same atom density per surface area as in a NiOOH layer.

The densities of states (DOS) were calculated for the pristine and the W-doped crystals, for bulk as well as for the (101) $Ni_{12}P_5$ slabs with Ni_3P-termination (see **Figure S22**). All the systems showed a similar DOS profile, where Ni3*d*-states dominate at the valence band and at the Fermi level (E_F). The only difference between the DOSs of the crystals and the slabs is in a slight downshift of E_F for the slabs, which was not dependent on the W content. Above 11% of W, W5*d*-states become visible near the E_F for the (101) slab, with a negligible contribution to the DOS. Therefore, W-doping does not influence the electric conductance of $Ni_{12}P_5$.

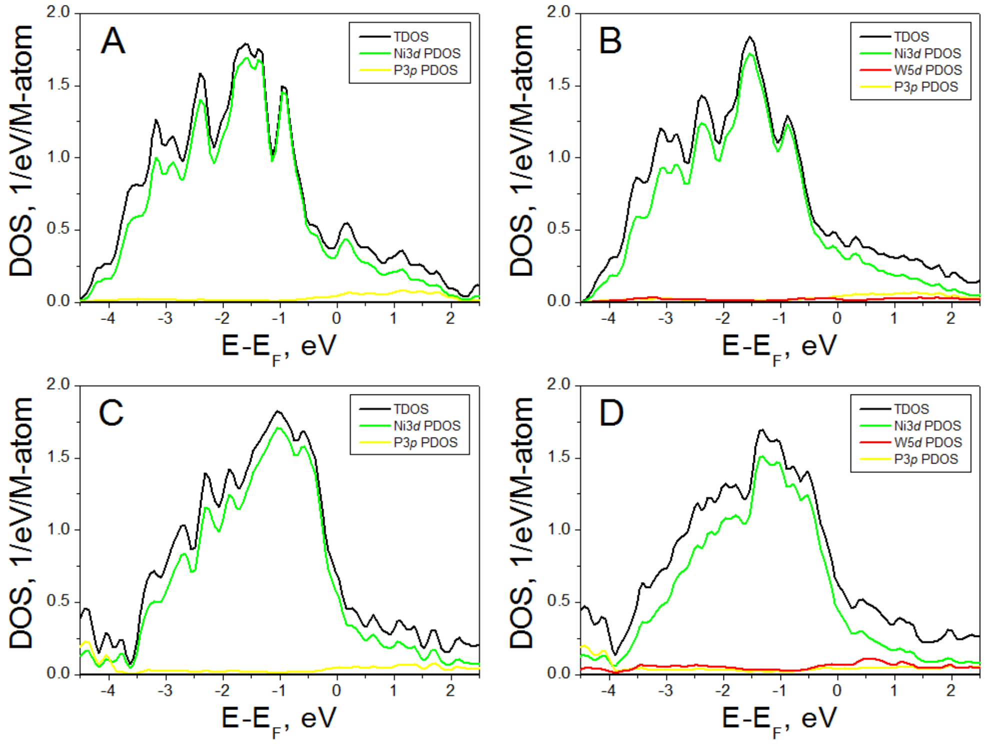

Figure S22. Total and selected partial electronic densities-of-states (DOS) for different $Ni_{12}P_5$ systems: (A) A bulk crystal; (B) A bulk crystal with 16% W-doping; (C) A slab with (101)Ni_3P-surface termination; (D) The same as (C) with 16% W-doping. DFT calculations.

On the thermodynamics of HER cycle using $Ni_{12}P_5$ and W-doped $Ni_{12}P_5$

The activity of an electrode material depends on both the number of catalytic sites with favorable hydrogen adsorption energy (ΔG_H) and on the electron transfer kinetics at the electrode/electrolyte interface. DFT calculations have been employed to distinguish the dominating factor in enhanced HER catalytic activity of W-$Ni_{12}P_5$ within cathode in alkaline media. The free energy ΔG_H of the adsorption of a single H atom on a substrate in vacuum

(relative to H_2 molecule and bare substrate) is a commonly used descriptor for the HER activity with a correction of +0.24 eV/H-atom.[28] Second correction accounting for the activity of hydrogen in 1 M hydroxide solution (alkaline) should be added to ΔG_H as +0.83 eV/H-atom. The perfect value of ΔG_H should be close to zero. The computed ΔG_H values for Ni- and P-sites at (101) Ni_3P-terminated surface of pristine $Ni_{12}P_5$ are equal to -0.03 eV/H-atom and +0.59 eV/H-atom, while those for Ni-, P- and W-sites at W-$Ni_{12}P_5$ are found as -0.05 eV/H-atom, +0.47 eV/H-atom and +0.11 eV/H-atom, respectively. According to these data, the Ni-sites act as the main catalytic sites in HER from an alkaline medium. Noteworthy, their reactivity from a thermodynamic point-of-view seems achieved already within the pristine compound and is not modulated further using W doping. Therefore, the enhanced activity of $Ni_{12}P_5$ catalyst upon W doping should be ascribed either to the increased number of catalytic sites (increased effective surface of the particles) or/and to improved kinetics; Specifically the kinetic energy barrier of the initial water dissociation step, which is considered as key parameter to enhance the alkaline HER catalytic activities.[28-30] The first option correlates with the experimental estimations of ESCA (section 3.1 in the main text). An increase in effective surface area may be also expected due to aforementioned thermodynamic destabilization of $Ni_{12}P_5$ lattice upon W doping. The second option is discussed below.

On the kinetics of water dissociation during alkaline HER using $Ni_{12}P_5$ and W-doped $Ni_{12}P_5$

Apart from thermodynamics consideration of H_2 release, the DFT calculations have been employed to estimate the free energies of the water adsorption and the water dissociation as the source of protons on pristine and W-doped $Ni_{12}P_5$. The same (101) $Ni_{12}P_5$ slabs as described above have served as the models of surface. Different adsorption sites have been considered at W-doped $Ni_{12}P_5$: adsorption on Ni atom surrounded by the only Ni and P atoms like in pristine $Ni_{12}P_5$; adsorption on Ni atom having a surface W atom in neighborhood; adsorption on W atom (**Figure S23**). The calculations show that, the W doping slightly promotes the water adsorption on Ni atoms remoted from the W impurity: adsorption energy becomes gainful for ~0.3 eV/H_2O-molecule, while adsorption on the W atoms and the Ni atoms nearby is not profitable, all compared to pristine $Ni_{12}P_5$ compound. In contrast, dissociation of water into surface H and OH species is drastically promoted by impurity atoms and their nearest environment with energy of dissociation on ~2 eV/H_2O-molecule lower, than for $Ni_{12}P_5$. At that, the energy barrier for reducing H gets higher or remains the same as

for $Ni_{12}P_5$. Such a diagram may be ascribed to poisoning of impurity environment blocked by dissociation products for the next round of dissociation. The Ni atoms remoted from impurities seem kinetically more attractive for performance: both the water dissociation energy step H--OH, and the energy barrier for H reduction are tolerably lower, than for $Ni_{12}P_5$ (-0.18 eV/H_2O and +0.66 eV/H-atom vs. -0.15/H_2O and +0.72 eV/H-atom, Figure S23.A-B).

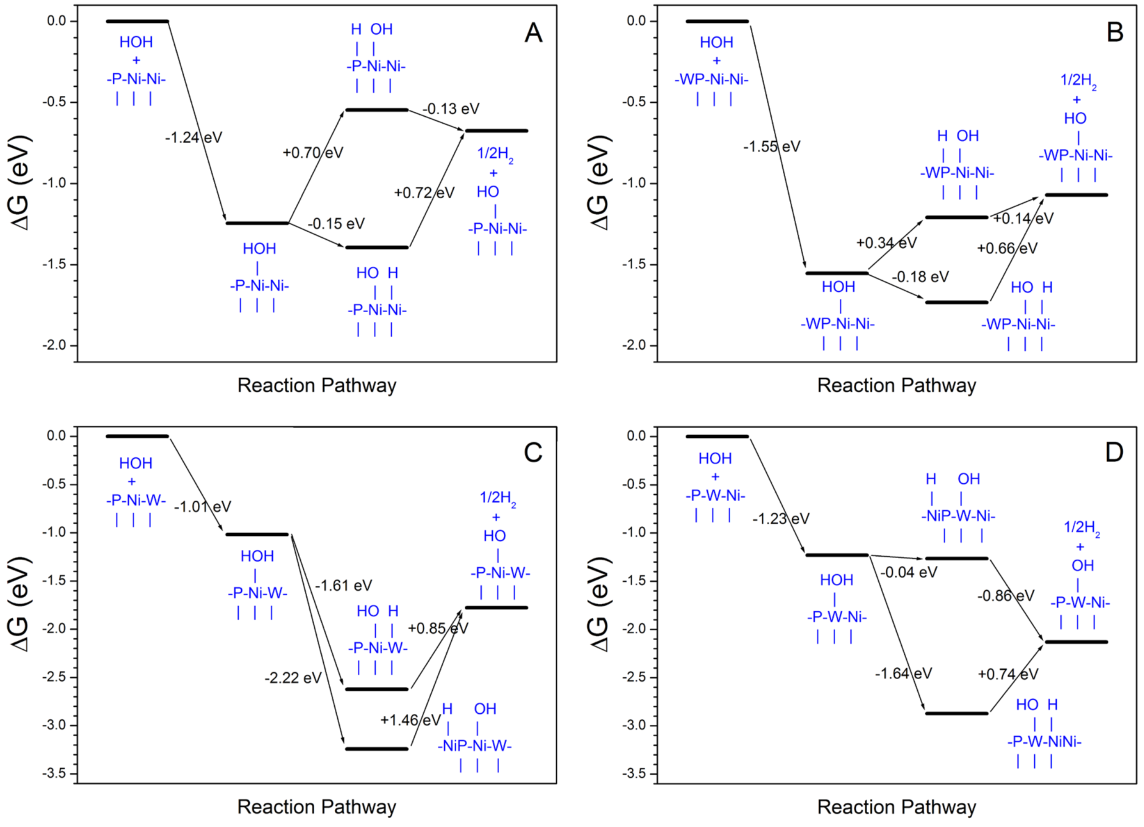

Figure S23. Free energy diagrams for the water dissociation and the hydrogen evolution on the (101)$Ni_{12}P_5$ surface (A) and on the (101)$W_{1.33}Ni_{10.67}P_5$ surface (B-D). Different adsorption sites have been considered for $W_{1.33}Ni_{10.67}P_5$: adsorption on Ni atom surrounded by the only Ni and P atoms like in pristine $Ni_{12}P_5$ (B); adsorption on Ni atom having a W atom in neighborhood (C); adsorption on W atom (D). Noticeably, P atom in (C) and (D) is found as extremely hostile for H atom: H atom appearing after water dissociation migrates exclusively to a Ni or W atom. DFT calculations.

Revealing the active substance in OER using $Ni_{12}P_5$ and W-doped $Ni_{12}P_5$

The behavior of $Ni_{12}P_5$ as anode material is more complex. The coverage of $Ni_{12}P_5$ by NiOOH within anode may make use of either the β-NiOOH or the γ-NiOOH phase. The β-NiOOH phase is loosely connected to the $Ni_{12}P_5$ substrate by a Ni-O bond, the Ni of the top Ni plane of $Ni_{12}P_5$

and the O from the NiOOH, as its lower surface is only partially populated by H **(Figure S24A)**. In contrast, γ-NiOOH, all the H atoms migrate to the top O plane, producing additional Ni-O bonds between the two materials and strengthening their cohesion **(Figure S24B)**. In addition, γ-NiOOH posseses also a dipole moment, which could further strengthen the adsorption. The calculations show that the formation of both interfaces is exothermic: -0.66 eV/NiOOH for γ-NiOOH and -0.31 eV/NiOOH for β-NiOOH, making the $Ni_{12}P_5$||γ-NiOOH interface more stable thermodynamically. This observation is opposite to the free-standing phases, where the polar γ-NiOOH layer is less stable. Therefore, at least 1-2 layers of γ-NiOOH can be thermodynamically stabilized in the vicinity of metallic $Ni_{12}P_5$ surface.

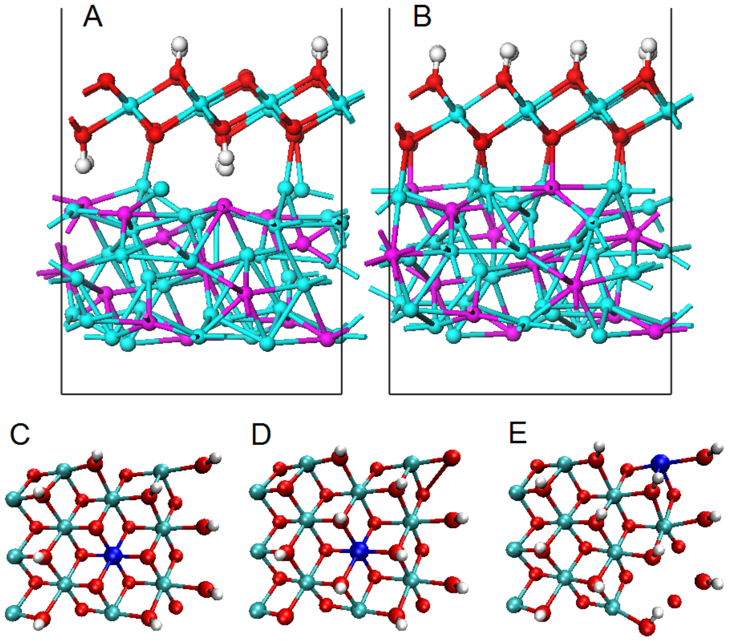

Figure S24. Ball-and-stick models for a single layer of NiOOH adsorbed on (101)$Ni_{12}P_5$ surface. Ni in cyan, P in violet, W in blue, O in red, and H in white. (A) β-NiOOH and (B) γ-NiOOH. W-doped γ-NiOOH with a single W atom may adopt three configurations: bare WO_6 octahedron (C), WO_6 with adsorbed H-atoms and three H-vacancies at the NiO_6H_3 octahedra (D) or a single Ni-vacancy (E). Structures C-E also appear in Figure 8 in the manuscript and are copied here for convenience.

We calculated the density of states of β-NiOOH and γ-NiOOH (**Figure S25**). Our calculations reproduced the DOS picture obtained in previous a report using the DFT and DFT+U methods for β-NiOOH as a metal-like compound with E_F in the local DOS minimum.[31] The former report as well as our calculations underestimate the band gap of NiOOH, compared with the experimental value. The calculations can be used to compare the partial Ni3*d*-DOS in both

compounds, which is important since the Ni3*d*-states near E_F are commonly considered as forming dative bonding to molecular adsorbates and by that they serve as charge transfer channels in redox reactions. For the free-standing NiOOH phases, the density of the Ni3*d*-states near E_F is 5 times larger in γ-NiOOH compared with β-NiOOH, and 2.5 times larger for $Ni_{12}P_5$|| γ-NiOOH compared with $Ni_{12}P_5$|| β -NiOOH.

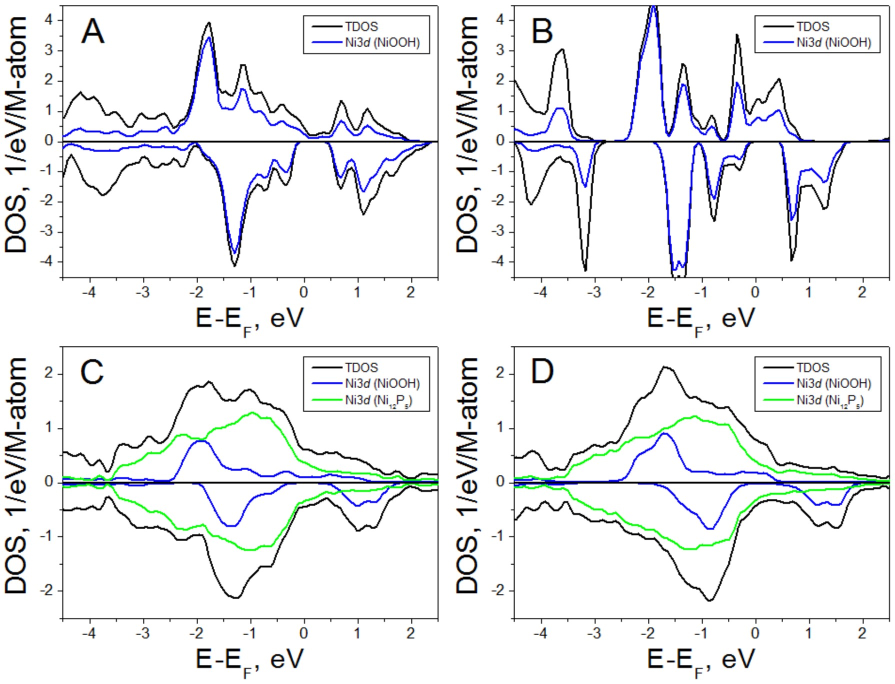

Figure S25. Total and selected partial electronic densities-of-states (DOS) of: (A) A single-layer of β-NiOOH; (B) A single layer of γ-NiOOH; (C) The (101)$Ni_{12}P_5$||(001)β-NiOOH interface; (D) The (101)$Ni_{12}P_5$||(001)γ-NiOOH interface.

While P is depleted from the surface during the catalytic measurements, Ni, O and W are present, proposing that the outer shell is W-doped γ-NiOOH, and hereafter, we focus exclusively on the influence of W-doping in γ-NiOOH. The oxidation states of W^{6+} and Ni^{3+} are different, and preservation of charge neutrality may be realized either by replacing three H vacancies or a single Ni-vacancy by one W ion (see **Figure 23C-E**). The chemical composition of the 3*a*×2*b* supercell of NiOOH is now $(Ni_{0.92}W_{0.08})OOH_{0.75}$ or $(Ni_{0.91}W_{0.09})OOH$. Since the composition is different than the pristine material, the relative stability of the mixed oxohydroxides was estimated relative to the total energy of the corresponding mixtures of NiOOH, WO_3 and H_2O. The calculations unveiled that substitutional W-doping is endothermic in both (Ni,W)OOH cases. The most stable variant is the formation of a bare

WO_6 octahedron within the NiOOH layer, with a formation energy of +0.07 eV/MOOH. The formation of H vacancies at the only NiO_6H_3 octahedra and the presence of WO_6H_3 octahedron is less favorable with formation energies of +0.12 eV/NiOOH. The least stable structure is (Ni,W)OOH with Ni-vacancy, with a formation energy of +0.17 eV/NiOOH.

The DOS near the Fermi level of a W-doped γ-NiOOH consist mostly of Ni3*d*-states, which are the main actors in chemical interactions or charge transfer across the interface to $(Ni,W)_{12}P_5$ substrate or to molecular adsorbates from solution. The analysis of the DOSs for all studied (Ni,W)OOH compounds allows to exclude a direct action of the W dopant during the reaction steps (**Figure S26**) since the prominent W5*d*-states are located ~0.7-1.5 eV above E_F, and cannot participate actively in the formation of covalent or dative bonding. The presence of $(Ni,W)_{12}P_5$ substrate does not change the general picture and is not contributing.

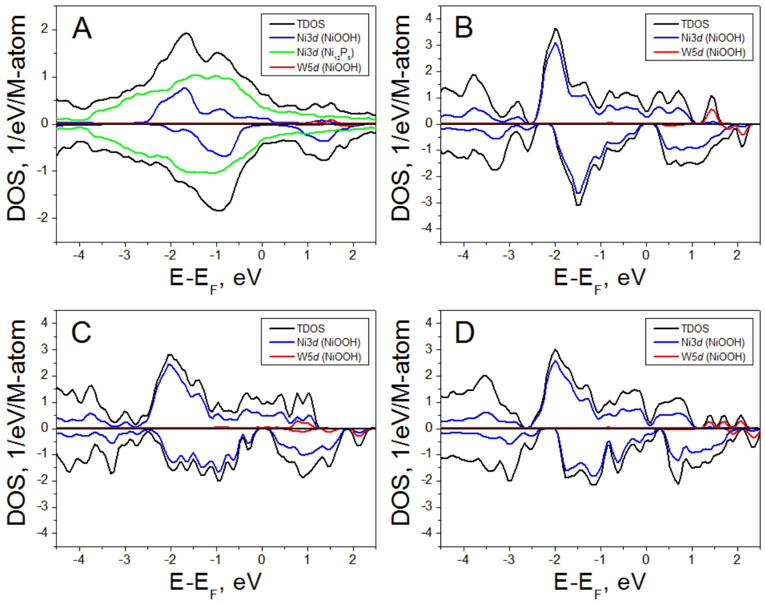

Figure S26. Total and selected partial electronic densities-of-states (DOS) for a single-layer of γ-(Ni,W)OOH with different structures: a bare WO_6 octahedron within a γ-(Ni,W)OOH layer, either on $Ni_{12}P_5$ (A) or as free-standing (B) – see the structure in Figure 16C; (C) γ-(Ni,W)OOH layer with adsorbed H-atoms and three H-vacancies at NiO_6H_3 octahedra, see the structure in Figure S16D; (D) γ-(Ni,W)OOH layer with single Ni-vacancy, see the structure in **Figure S24E**.

Table S4. Variation in the barrier energies ΔG for the OER steps in the model of the single-site associative reaction mechanism (reaction steps I-VI, see **Figure 7** in the main text), employing different γ-NiOOH catalysts doped by single W atoms: (A) pristine undoped layer; (B) layer containing the bare WO_6 octahedron (reaction site); (C) layer containing the WO_6H_3 octahedron (reaction site) and three H-vacancies at NiO_6H_3 octahedra; (D) layer containing single Ni-vacancy near the WO_6H_3 octahedron (reaction site); (E) layer containing single Ni-vacancy (reaction site) near the WO_6H_3 octahedron. DFT calculations.

step	ΔG, eV				
	(A)	(B)	(C)	(D)	(E)
I-II	-1.84	+0.07	-2.10	-1.32	-1.38
III	+0.35	+1.23	+1.76	+0.65	+1.15
IV	+3.28	+2.75	+1.91	+2.94	+2.73
V	+1.81	+1.11	+1.85	+1.86	+1.64
VI	+1.33	-0.23	+1.51	+0.80	+0.79
Sum	+4.93	+4.93	+4.93	+4.93	+4.93

References:

1. Meiron, O. E.; Kuraganti, V.; Hod, I.; Bar-Ziv, R.; Bar-Sadan, M., Improved Catalytic Activity of $Mo_{1-X}W_xSe_2$ Alloy Nanoflowers Promotes Efficient Hydrogen Evolution Reaction in Both Acidic and Alkaline Aqueous Solutions. *Nanoscale* **2017,** *9* (37), 13998-14005.
2. Ranaweera, C. K.; Zhang, C.; Bhoyate, S.; Kahol, P. K.; Ghimire, M.; Mishra, S. R.; Perez, F.; Gupta, B. K.; Gupta, R. K., Flower-Shaped Cobalt Oxide Nano-Structures as an Efficient, Flexible and Stable Electrocatalyst for the Oxygen Evolution Reaction. *Mater. Chem. Front.* **2017,** *1* (8), 1580-1584.
3. Xin, W.; Jiang, W.-J.; Lian, Y.; Li, H.; Hong, S.; Xu, S.; Yan, H.; Hu, J.-S., Nis_2 Nanodotted Carnation-Like CoS_2 for Enhanced Electrocatalytic Water Splitting. *Chem. Commun.* **2019,** *55* (26), 3781-3784.
4. Wei, X.; Zhang, Y.; He, H.; Peng, L.; Xiao, S.; Yao, S.; Xiao, P., Carbon-Incorporated Porous Honeycomb NiCoFe Phosphide Nanospheres Derived from a MOF Precursor for Overall Water Splitting. *Chem. Commun.* **2019,** *55* (73), 10896-10899.
5. Kim, M. S.; Abbas, M. A.; Thota, R.; Bang, J. H., Thermally Induced Top-Down Nanostructuring for the Synthesis of a Core/Shell-Structured CoO/CoS_x Electrocatalyst. *J. Mater. Chem. A* **2019,** *7* (46), 26557-26565.
6. Wang, Q.; Zhao, H.; Li, F.; She, W.; Wang, X.; Xu, L.; Jiao, H., Mo-Doped Ni_2p Hollow Nanostructures: Highly Efficient and Durable Bifunctional Electrocatalysts for Alkaline Water Splitting. *J. Mater. Chem. A* **2019,** *7* (13), 7636-7643.

7. Xu, Y.; Duan, S.; Li, H.; Yang, M.; Wang, S.; Wang, X.; Wang, R., Au/$Ni_{12}P_5$ Core/Shell Single-Crystal Nanoparticles as Oxygen Evolution Reaction Catalyst. *Nano Res.* **2017,** *10* (9), 3103-3112.
8. Xue, Z.-H.; Su, H.; Yu, Q.-Y.; Zhang, B.; Wang, H.-H.; Li, X.-H.; Chen, J.-S., Janus Co/CoP Nanoparticles as Efficient Mott-Schottky Electrocatalysts for Overall Water Splitting in Wide Ph Range. *Adv. Energy Mater.* **2017,** *7* (12), 1602355.
9. Sankar, S.; Sugawara, Y.; Assa Aravindh, S.; Jose, R.; Tamaki, T.; Anilkumar, G. M.; Yamaguchi, T., Tuning Palladium Nickel Phosphide toward Efficient Oxygen Evolution Performance. *ACS Appl. Energy Mater.* **2020,** *3* (1), 879-888.
10. Zhou, G.; Li, M.; Li, Y.; Dong, H.; Sun, D.; Liu, X.; Xu, L.; Tian, Z.; Tang, Y., Regulating the Electronic Structure of Cop Nanosheets by O Incorporation for High-Efficiency Electrochemical Overall Water Splitting. *Adv. Funct. Mater.* **2019,** *30* (7), 1905252.
11. Zhang, Y.; Li, N.; Zhang, Z.; Li, S.; Cui, M.; Ma, L.; Zhou, H.; Su, D.; Zhang, S., Programmable Synthesis of Multimetallic Phosphide Nanorods Mediated by Core/Shell Structure Formation and Conversion. *J. Am. Chem. Soc.* **2020,** *142* (18), 8490-8497.
12. Liang, X.; Zheng, B.; Chen, L.; Zhang, J.; Zhuang, Z.; Chen, B., Mof-Derived Formation of Ni_2P-CoP Bimetallic Phosphides with Strong Interfacial Effect toward Electrocatalytic Water Splitting. *ACS Appl. Mater. Interfaces* **2017,** *9* (27), 23222-23229.
13. Chen, H.; Huang, X.; Zhou, L.-J.; Li, G.-D.; Fan, M.; Zou, X., Electrospinning Synthesis of Bimetallic Nickel–Iron Oxide/Carbon Composite Nanofibers for Efficient Water Oxidation Electrocatalysis. *ChemCatChem* **2016,** *8* (5), 992-1000.
14. Ledendecker, M.; Krick Calderón, S.; Papp, C.; Steinrück, H.-P.; Antonietti, M.; Shalom, M., The Synthesis of Nanostructured Ni_5P_4 Films and Their Use as a Non-Noble Bifunctional Electrocatalyst for Full Water Splitting. *Angew. Chem. Int. Ed.* **2015,** *54* (42), 12361-12365.
15. Yeo, B. S.; Bell, A. T., In Situ Raman Study of Nickel Oxide and Gold-Supported Nickel Oxide Catalysts for the Electrochemical Evolution of Oxygen. *J. Phys. Chem. C* **2012,** *116* (15), 8394-8400.
16. Huang, J.; Li, Y.; Zhang, Y.; Rao, G.; Wu, C.; Hu, Y.; Wang, X.; Lu, R.; Li, Y.; Xiong, J., Identification of Key Reversible Intermediates in Self-Reconstructed Nickel-Based Hybrid Electrocatalysts for Oxygen Evolution. *Angew. Chem. Int. Ed.* **2019,** *58* (48), 17458-17464.
17. Lo, Y. L.; Hwang, B. J., In Situ Raman Studies on Cathodically Deposited Nickel Hydroxide Films and Electroless Ni−P Electrodes in 1 M Koh Solution. *Langmuir* **1998,** *14* (4), 944-950.
18. Yang, C. C.; Zai, S. F.; Zhou, Y. T.; Du, L.; Jiang, Q., Fe_3c-Co Nanoparticles Encapsulated in a Hierarchical Structure of N-Doped Carbon as a Multifunctional Electrocatalyst for ORR, OER, and HER. *Adv. Func. Mater.* **2019,** *29* (27), 1901949.
19. Yu, L.; Xiao, Y.; Luan, C.; Yang, J.; Qiao, H.; Wang, Y.; Zhang, X.; Dai, X.; Yang, Y.; Zhao, H., Cobalt/Molybdenum Phosphide and Oxide Heterostructures Encapsulated in N-Doped Carbon Nanocomposite for Overall Water Splitting in Alkaline Media. *ACS Appl. Mater. Interfaces* **2019,** *11* (7), 6890-6899.
20. Yang, Y.; Lin, Z.; Gao, S.; Su, J.; Lun, Z.; Xia, G.; Chen, J.; Zhang, R.; Chen, Q., Tuning Electronic Structures of Nonprecious Ternary Alloys Encapsulated in Graphene Layers for Optimizing Overall Water Splitting Activity. *ACS Catal.* **2017,** *7* (1), 469-479.
21. Gao, X.; Zhang, H.; Li, Q.; Yu, X.; Hong, Z.; Zhang, X.; Liang, C.; Lin, Z., Hierarchical $Nico_2o_4$ Hollow Microcuboids as Bifunctional Electrocatalysts for Overall Water-Splitting. *Angew. Chem. Int. Ed.* **2016,** *55* (21), 6290-6294.
22. Ordejón, P.; Artacho, E.; Soler, J. M., Self-Consistent Order-N Density-Functional Calculations for Very Large Systems. *Phys. Rev. B* **1996,** *53* (16), R10441.
23. Troullier, N.; Martins, J. L., Efficient Pseudopotentials for Plane-Wave Calculations. *Phys. Rev. B* **1991,** *43* (3), 1993-2006.
24. Hu, J.; Chen, W.; Zhao, X.; Cao, X.; Zhu, J.; Chen, Z., Stable Active Sites on $Ni_{12}P_5$ Surfaces for the Hydrogen Evolution Reaction. *Energy Technology* **2019,** *7* (6), 1900013.

25. Duan, S.; Wang, R., Au/$Ni_{12}P_5$ Core/Shell Nanocrystals from Bimetallic Heterostructures: In Situ Synthesis, Evolution and Supercapacitor Properties. *NPG Asia Mater.* **2014,** *6* (9), e122-e122.
26. Zhang, W.; Zheng, J.; Gu, X.; Tang, B.; Li, J.; Wang, X., Facile Synthesis, Characterization and Dft Studies of a Nanostructured Nickel–Molybdenum–Phosphorous Planar Electrode as an Active Electrocatalyst for the Hydrogen Evolution Reaction. *Nanoscale* **2019,** *11* (19), 9353-9361.
27. Chen, Z.; Shan, A.; Cui, Y.; Wang, R.; Chen, C., Catalysis of Hydrogen Evolution Reaction by $Ni_{12}P_5$ Single Crystalline Nanoplates and Spherical Nanoparticles. *CrystEngComm* **2019,** *21* (2), 228-235.
28. Mahmood, N.; Yao, Y.; Zhang, J.-W.; Pan, L.; Zhang, X.; Zou, J.-J., Electrocatalysts for Hydrogen Evolution in Alkaline Electrolytes: Mechanisms, Challenges, and Prospective Solutions. *Adv. Sci.* **2018,** *5* (2), 1700464.
29. Danilovic, N.; Subbaraman, R.; Strmcnik, D.; Chang, K.-C.; Paulikas, A. P.; Stamenkovic, V. R.; Markovic, N. M., Enhancing the Alkaline Hydrogen Evolution Reaction Activity through the Bifunctionality of $Ni(OH)_2$/Metal Catalysts. *Angew. Chem. Int. Ed.* **2012,** *51* (50), 12495-12498.
30. Farinazzo Bergamo Dias Martins, P.; Papa Lopes, P.; Ticianelli, E. A.; Stamenkovic, V. R.; Markovic, N. M.; Strmcnik, D., Hydrogen Evolution Reaction on Copper: Promoting Water Dissociation by Tuning the Surface Oxophilicity. *Electrochem. commun.* **2019,** *100*, 30-33.
31. Tkalych, A. J.; Yu, K.; Carter, E. A., Structural and Electronic Features of B-$Ni(OH)_2$ and B-NiOOH from First Principles. *J. Phys. Chem. C* **2015,** *119* (43), 24315-24322.
